# Microglia-targeted delivery of MCC950 suppresses NLRP3–IL-18-mediated neuroinflammation and retinal injury in retinal vein occlusion

**DOI:** 10.1016/j.bioactmat.2026.10.022

**Published:** 2026-10-22

**Authors:** Xiao Xue, Huimin Jiang, Weiyue Zhang, Yifan Zhou, Hui Li, Chengbin Zhou, Yuxin Chen, Wei Ma, Yuan Gao, Xunming Ji, Chen Zhou, Xuxiang Zhang

**Affiliations:** a Department of Ophthalmology, Xuanwu Hospital, Capital Medical University, Beijing, 100053, China; b Beijing Institute of Brain Disorders, Laboratory of Brain Disorders, Ministry of Science and Technology, Collaborative Innovation Center for Brain Disorders, Beijing Advanced Innovation Center for Big Data-based Precision Medicine, Capital Medical University, Beijing, 100069, China; c Beijing Advanced Innovation Center for Big Data-Based Precision Medicine, School of Biological Science and Medical Engineering, Beihang University, Beijing, 100191, China; d Department of Neurology, Xuanwu Hospital, Capital Medical University, Beijing, 100053, China; e Department of Neurosurgery, Ruijin Hospital, Shanghai Jiao Tong University School of Medicine, Shanghai, 200025, China; f Neuro Cardio Vascular Disease Center, Xuanwu Hospital, Capital Medical University, Beijing, 100053, China

**Keywords:** Retinal vein occlusion, Microglia, NLRP3 inflammasome, Targeted drug delivery, Nanoliposomes

## Abstract

Retinal vein occlusion (RVO) causes retinal ischemia, macular edema, and vision loss, yet current anti-vascular endothelial growth factor (anti-VEGF) therapies primarily limit vascular leakage and leave ischemia-driven neuroinflammation insufficiently controlled. Here, we reveal a critical role for microglia-associated NLRP3–IL-18 signaling in amplifying inflammation during early RVO. Bulk retinal RNA sequencing showed broad innate immune activation, whereas single-cell transcriptomic profiling and spatial protein analysis identified prominent inflammasome-related changes in retinal microglia. Constitutive global deletion of *Nlrp3* or *Il18* attenuated microglial activation and preserved retinal structure and electrophysiological function. To translate this mechanistic insight into a targeted anti-inflammatory strategy, we engineered MG1-functionalized, MCC950-loaded nanoliposomes for microglia-targeted NLRP3 inhibition. MG1 functionalization promoted preferential nanoparticle uptake by activated microglia and suppressed NLRP3–IL-18 signaling more effectively than free MCC950 or non-targeted nanoliposomes. In experimental RVO, a single intravitreal dose administered 6 h after induction alleviated retinal inflammation and edema while preserving retinal electrophysiological responses. These findings establish microglia-associated NLRP3–IL-18 signaling as a therapeutic target in early RVO and support MG1-MCC950 nanoliposomes as a bioactive nanotherapeutic platform for RVO.

## Highlights

• Single-cell profiling highlights microglial inflammatory responses in RVO.
• Global *Nlrp3* or *Il18* deletion limits retinal injury and preserves ERG responses.
• MG1 functionalization promotes preferential nanoliposome uptake by retinal microglia.
• MG1-MCC950 nanoliposomes suppress NLRP3-IL-18 signaling and retinal injury.
• MG1-MCC950 nanoliposomes outperform free MCC950 after RVO.

## Graphical abstract

**Figure:**
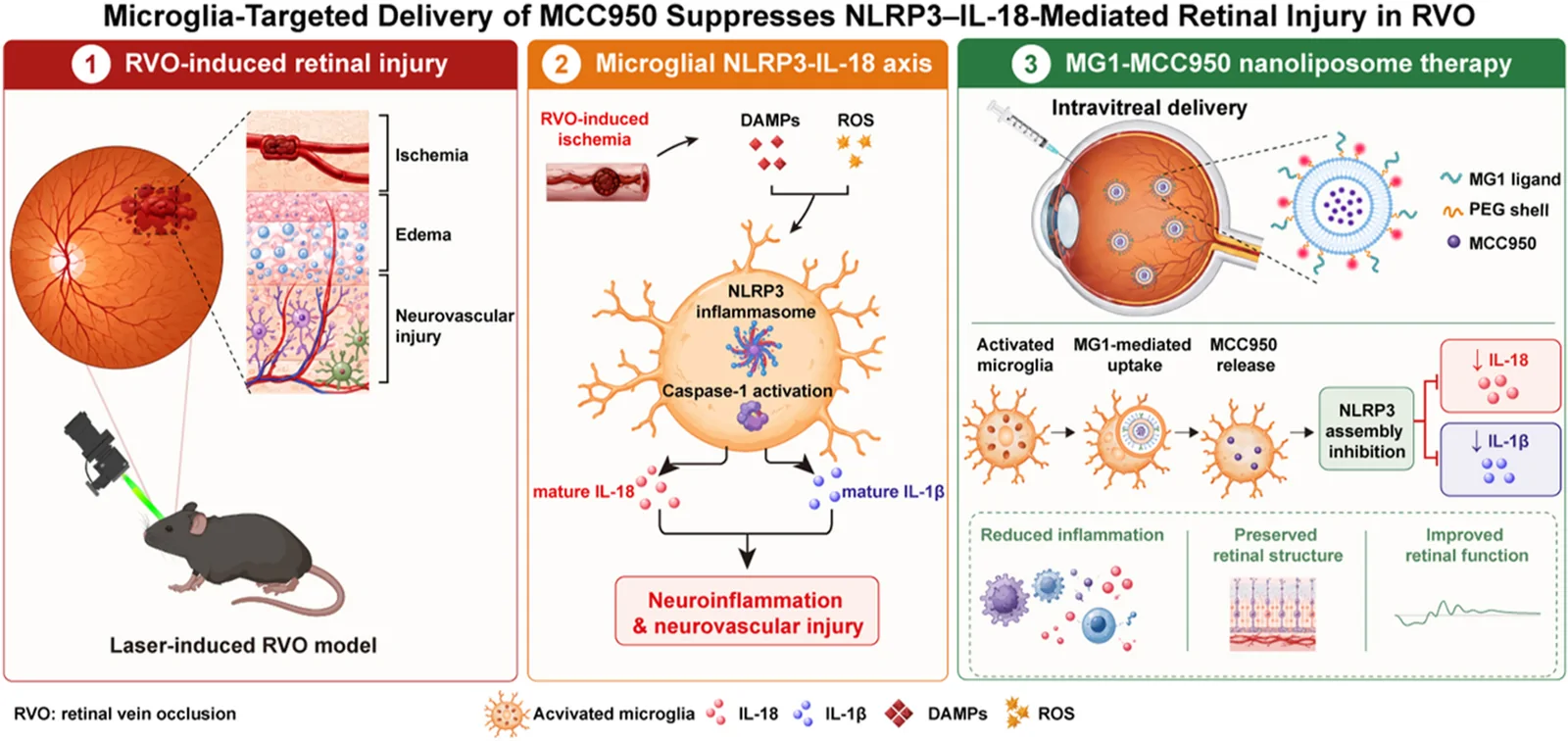

## 1 Introduction

Retinal vein occlusion (RVO) is a major cause of visual morbidity and one of the most common retinal vascular disorders worldwide [1]. Impaired venous outflow and hemodynamic congestion trigger blood–retinal barrier disruption, macular edema, retinal ischemia, and progressive neurovascular dysfunction [2], [3]. Although anti-VEGF agents and intravitreal corticosteroids have substantially improved clinical management, important therapeutic limitations remain [4], [5]. Many patients experience recurrent or treatment-refractory macular edema, require frequent intravitreal injections, or continue to lose neuroretinal tissue despite anatomical improvement [6], [7]. Current standard-of-care therapies primarily target vascular leakage, whereas ischemia-driven neuroinflammatory injury remains insufficiently addressed [8], [9].

Sterile inflammation is increasingly recognized as an active contributor to retinal injury after venous occlusion. Following RVO, the hypoxic and stressed retinal microenvironment induces inflammatory mediators, including IL-6, IL-8, and MCP-1, whose intraocular levels have been associated with disease activity and macular edema severity [10], [11]. Microglia, the resident innate immune cells of the retina, are well positioned to sense ischemic stress and orchestrate secondary inflammatory responses. Under pathological conditions, microglia rapidly transition from a homeostatic surveillance state to activated phenotypes, migrate toward injured regions, and release cytokines and chemokines that amplify neurovascular dysfunction [12], [13]. Experimental evidence from ischemic retinal injury models further suggests that microglial activation contributes to tissue damage rather than merely reflecting injury [14], [15], [16]. However, the molecular checkpoints that initiate and sustain microglia-associated inflammatory amplification during the early phase of RVO remain incompletely defined [17], [18], [19].

The NLRP3 inflammasome is a canonical innate immune sensor that links cellular stress and danger-associated molecular patterns to inflammatory cytokine maturation. Upon activation, NLRP3 promotes caspase-1-dependent processing of IL-1β and IL-18, thereby converting sterile injury signals into a propagated inflammatory response [20], [21]. NLRP3 signaling has been implicated in several retinal diseases, including diabetic retinopathy, age-related macular degeneration, and ischemic retinal injury [22], [23], [24], [25]. Nevertheless, its cell-type-enriched contribution to acute RVO-associated retinal injury remains unclear. Although IL-18 upregulation has been reported in experimental RVO, whether NLRP3 and IL-18 contribute functionally to acute retinal injury, and whether this signaling is preferentially associated with microglia, remain unclear.

These knowledge gaps provided a rationale for evaluating NLRP3 inhibition in RVO. MCC950 is a well-characterized small-molecule inhibitor of NLRP3 inflammasome activation [26]. However, free MCC950 lacks intrinsic cell-type specificity within the inflamed retina. This limitation is particularly relevant because disruption of the blood–retinal barrier may expose multiple resident retinal and infiltrating immune cell populations to intravitreally administered drugs. Given the prominent inflammatory response of microglia after venous occlusion, we hypothesized that preferential delivery to activated microglia could increase local drug exposure and enhance NLRP3 inhibition. MG1 is a nine-amino-acid peptide with the sequence CHHSSSARC that was originally identified through phage-display screening and reported to exhibit preferential affinity for pro-inflammatory microglia [27]. These properties provided the rationale for its use as the targeting ligand in the present nanoliposomal platform.

Here, we developed a mechanism-guided microglia-targeted nanoliposomal platform for inflammasome-directed therapy in RVO. We combined bulk retinal RNA sequencing, single-cell transcriptomics, spatial protein analysis, constitutive global *Nlrp3* and *Il18* knockout models, and multimodal retinal phenotyping to characterize the cellular distribution and functional relevance of NLRP3–IL-18 signaling in early RVO injury. Guided by these mechanistic findings, we engineered MG1-functionalized, MCC950-loaded nanoliposomes to enhance NLRP3 inhibitor delivery to activated microglia. We characterized their physicochemical properties and evaluated their preferential microglial uptake, therapeutic efficacy, and preliminary biocompatibility. Together, this work integrates mechanistic discovery, cell-enriched drug delivery, and biomaterial design into a targeted anti-inflammatory strategy for retinal protection after RVO.

## 2 Results

### 2.1 Establishment and validation of the experimental RVO model

Experimental RVO was induced by Rose Bengal-sensitized laser photothrombosis. In the time-course cohort shown in Fig. 1, infrared reflectance (IR) fundus imaging, fundus fluorescein angiography (FFA), and optical coherence tomography (OCT) were performed on day 0 (D0) after RVO induction. IR fundus imaging, OCT, and electroretinography (ERG) were subsequently performed from day 1 (D1) to day 3 (D3) (Fig. 1A).

**Fig. 1:**
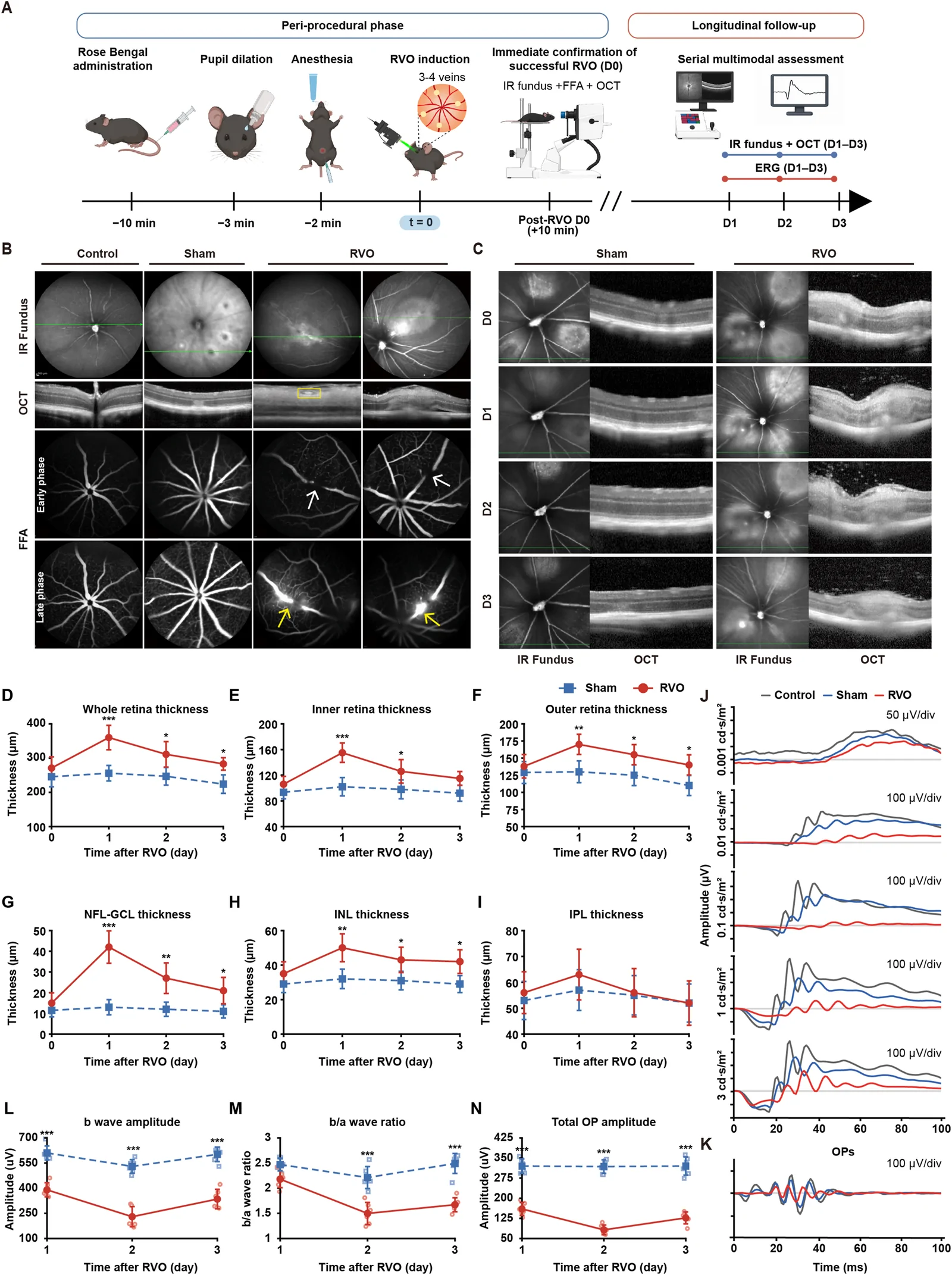
**Establishment and temporal characterization of experimental retinal vein occlusion (RVO) in mice.** (A) Schematic of Rose Bengal-sensitized laser photothrombosis, including Rose Bengal administration, pupil dilation, anesthesia, laser-induced venous occlusion, and angiographic assessment approximately 10 min after irradiation. (B) Representative infrared reflectance (IR) fundus images, optical coherence tomography (OCT) B-scans, and early- and late-phase fundus fluorescein angiography (FFA) from control, sham, and RVO eyes. The yellow box marks a laser-treated venous segment on OCT. Early-phase FFA showed delayed or absent filling of treated venous segments and impaired downstream perfusion (white arrows), whereas late-phase FFA showed vascular leakage after RVO (yellow arrows). (C) Serial IR fundus images and OCT B-scans acquired from D0 through D3, showing acute retinal thickening that was greatest on D1 and partially subsided over D2–D3. (D–I) Quantification of whole retinal, inner retinal, outer retinal, nerve fiber layer–ganglion cell layer (NFL–GCL), inner nuclear layer (INL), and inner plexiform layer (IPL) thickness, respectively. (J) Representative scotopic electroretinography (ERG) waveforms recorded at the indicated flash intensities. (K) Representative oscillatory potential (OP) traces recorded at 3.0 cd s/m^2^. (L–N) Quantification of b wave amplitude, b/a wave ratio, and total OP amplitude from responses elicited at 3.0 cd s/m^2^. Data in D–I and L–N are presented as the mean ± SD; *n* = 6 animals per group. Statistical comparisons were performed using two-way repeated-measures ANOVA followed by Šídák's multiple-comparisons test. *P < 0.05, **P < 0.01, and ***P < 0.001. D0, day 0; D1, day 1; D2, day 2; D3, day 3.

Across all experimental series, 231 mice entered the RVO-induction workflow. Of these, 192 met the prespecified eligibility criteria during direct funduscopic reassessment approximately 10 min after irradiation, yielding an overall procedural eligibility rate of 83.1%. Of the 39 mice excluded before downstream assessment, 21 failed to exhibit or maintain the required funduscopic signs of impaired venous flow, including eyes in which visible venous blood-column continuity was restored early; 4 developed anesthesia-related complications or hypothermia; 7 had persistent lens or other ocular media opacities that prevented reliable fundus assessment; 5 developed extensive preretinal hemorrhage that precluded reliable fundus or structural evaluation; and 2 sustained accidental injury to an adjacent retinal artery. All eligibility decisions were made before functional, molecular, flow-cytometric, or histological outcomes were assessed.

IR fundus imaging delineated retinal vascular morphology and the laser-treated sites. FFA showed largely preserved vascular filling in control and sham eyes. In RVO eyes, early-phase FFA revealed delayed or absent filling of the treated venous segments and impaired downstream perfusion, whereas late-phase FFA showed marked vascular leakage (Fig. 1B). Serial IR fundus imaging documented the evolution of fundus changes, whereas OCT demonstrated acute regional retinal thickening that peaked on D1 and partially subsided over day 2 (D2) to D3 (Fig. 1C). Whole retinal thickness increased from approximately 270 μm on post-induction D0 to approximately 355 μm on D1 and then partially declined over D2–D3, whereas retinal thickness in sham eyes remained comparatively stable (Fig. 1D). Retinal thickening involved both the inner and outer retina and was particularly pronounced in the nerve fiber layer–ganglion cell layer (NFL–GCL) and inner nuclear layer (INL), whereas the inner plexiform layer (IPL) showed comparatively modest changes (Fig. 1E–I). OCT B-scans used for quantification were acquired tangentially, immediately outside the visible photocoagulation margin rather than through the lesion core.

Representative scotopic ERG responses were attenuated across increasing flash intensities in RVO eyes compared with control and sham eyes, and oscillatory potentials (OPs) were similarly reduced (Fig. 1J and K). Quantification across D1–D3 confirmed reductions in b wave amplitude, the b/a wave ratio, and OP amplitude, with the greatest impairment observed on D2 (Fig. 1L–N). These findings characterize an acute experimental RVO phenotype marked by regional retinal thickening with partial resolution and sustained retinal electrophysiological impairment during the observation period.

### 2.2 Bulk retinal transcriptomics identifies an early inflammatory response after RVO

Bulk RNA sequencing was performed on retinas collected 48 h after RVO induction to characterize the transcriptional response during the acute injury phase. Exploratory bulk RNA-seq analysis identified 460 candidate genes with higher estimated abundance and 332 with lower estimated abundance in the pooled RVO library relative to the pooled Control library. Heatmap visualization showed distinct expression patterns between the two pooled libraries (Fig. 2A and B). Kyoto Encyclopedia of Genes and Genomes (KEGG) and Gene Ontology (GO) biological process enrichment analyses showed that the upregulated genes were predominantly associated with immune and inflammatory processes, including complement and coagulation cascades, cytokine signaling, leukocyte-mediated immunity, antigen processing and presentation, and NOD-like receptor signaling (Fig. 2C and D).

**Fig. 2:**
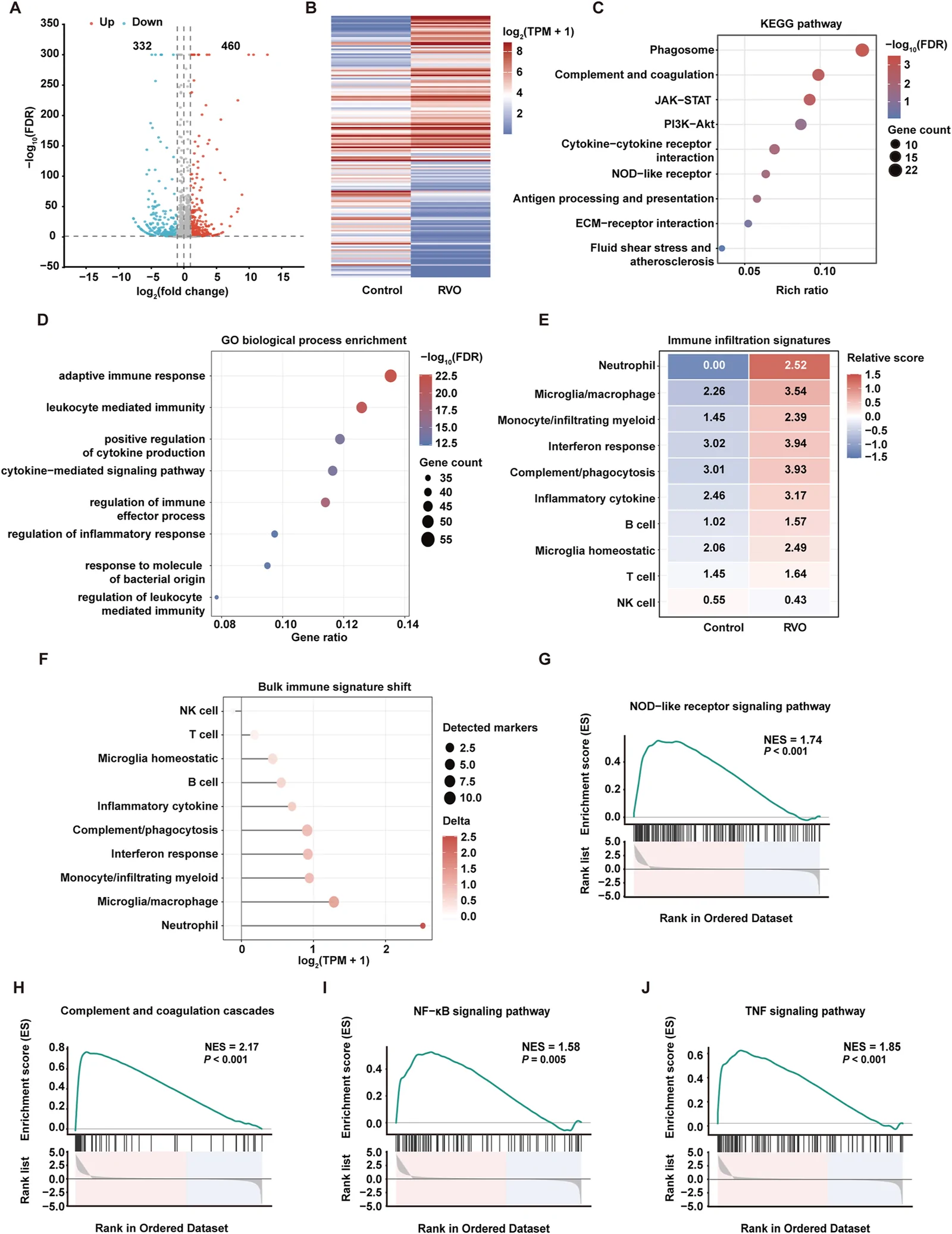
**Bulk retinal transcriptomics reveals early immune and inflammatory pathway activation after RVO.** (A) Volcano plot showing 460 candidate genes with higher estimated abundance and 332 with lower estimated abundance in the pooled RVO library relative to the pooled Control library 48 h after RVO induction. Exploratory candidates were selected using |log_2_ fold change| ≥ 1 and FDR < 0.05. Gene-wise *P* values were calculated using two-sided Fisher's exact tests and adjusted using the Benjamini–Hochberg procedure. Genes with higher and lower estimated abundance are shown in red and blue, respectively. (B) Heatmap of candidate genes identified by the exploratory bulk RNA-seq analysis, showing their expression patterns in the pooled Control and RVO libraries as log_2_(TPM + 1). (C, D) Kyoto Encyclopedia of Genes and Genomes (KEGG) pathway and Gene Ontology (GO) biological-process enrichment analyses, respectively. Point size represents gene count, and color represents −log_10_(FDR). (E) Heatmap of marker-based immune-signature scores in Control and RVO retinas. (F) Changes in immune-signature scores between RVO and Control, including neutrophil, microglia/macrophage, monocyte/infiltrating-myeloid, interferon-response, complement/phagocytosis, inflammatory-cytokine, and other immune-related signatures. Point size represents the number of detected marker genes, and color represents the change in signature score relative to Control. (G–J) Gene set enrichment analysis (GSEA) of NOD-like receptor signaling, complement and coagulation cascades, NF-κB signaling, and TNF signaling, respectively. Normalized enrichment scores (NES) and *P* values are shown in the corresponding panels. Each group comprised one pooled library prepared from 10 retinas from five animals. FDR, false discovery rate; TPM, transcripts per million.

Consistent with these enrichment results, marker-based immune-signature analysis showed higher scores for neutrophil, microglia/macrophage, monocyte/infiltrating-myeloid, interferon-response, complement/phagocytosis, and inflammatory-cytokine modules after RVO (Fig. 2E and F). Gene set enrichment analysis (GSEA) confirmed positive enrichment of NOD-like receptor signaling (normalized enrichment score [NES] = 1.74, *P* < 0.001), complement and coagulation cascades (NES = 2.17, *P* < 0.001), NF-κB signaling (NES = 1.58, *P* = 0.005), and TNF signaling (NES = 1.85, *P* < 0.001) (Fig. 2G–J). Together, the bulk data indicate that early RVO is accompanied by a broad innate-immune response, with complement activation, cytokine signaling, and NOD-like receptor programs emerging as prominent molecular features.

### 2.3 Single-cell transcriptomics identifies microglia as a prominent inflammatory compartment after RVO

Single-cell RNA sequencing was performed 48 h after RVO induction to characterize the cellular distribution of the retinal inflammatory response. After quality control, 6616 Control cells and 9134 RVO cells were retained. Unsupervised clustering resolved the major retinal cell populations, and marker heatmap and dot-plot analyses confirmed their annotation using canonical retinal markers (Fig. 3A–C and Fig. S1A–C). Control and RVO cells formed a shared retinal atlas, enabling direct comparison of cell-type composition and transcriptional state (Fig. S1D–E). Among the annotated populations, microglia exhibited the most prominent descriptive difference between the two pooled libraries: 87.2% of cells in the microglial cluster were derived from RVO retinas, whereas most neuronal and glial populations showed a more balanced Control/RVO distribution (Fig. 3D).

**Fig. 3:**
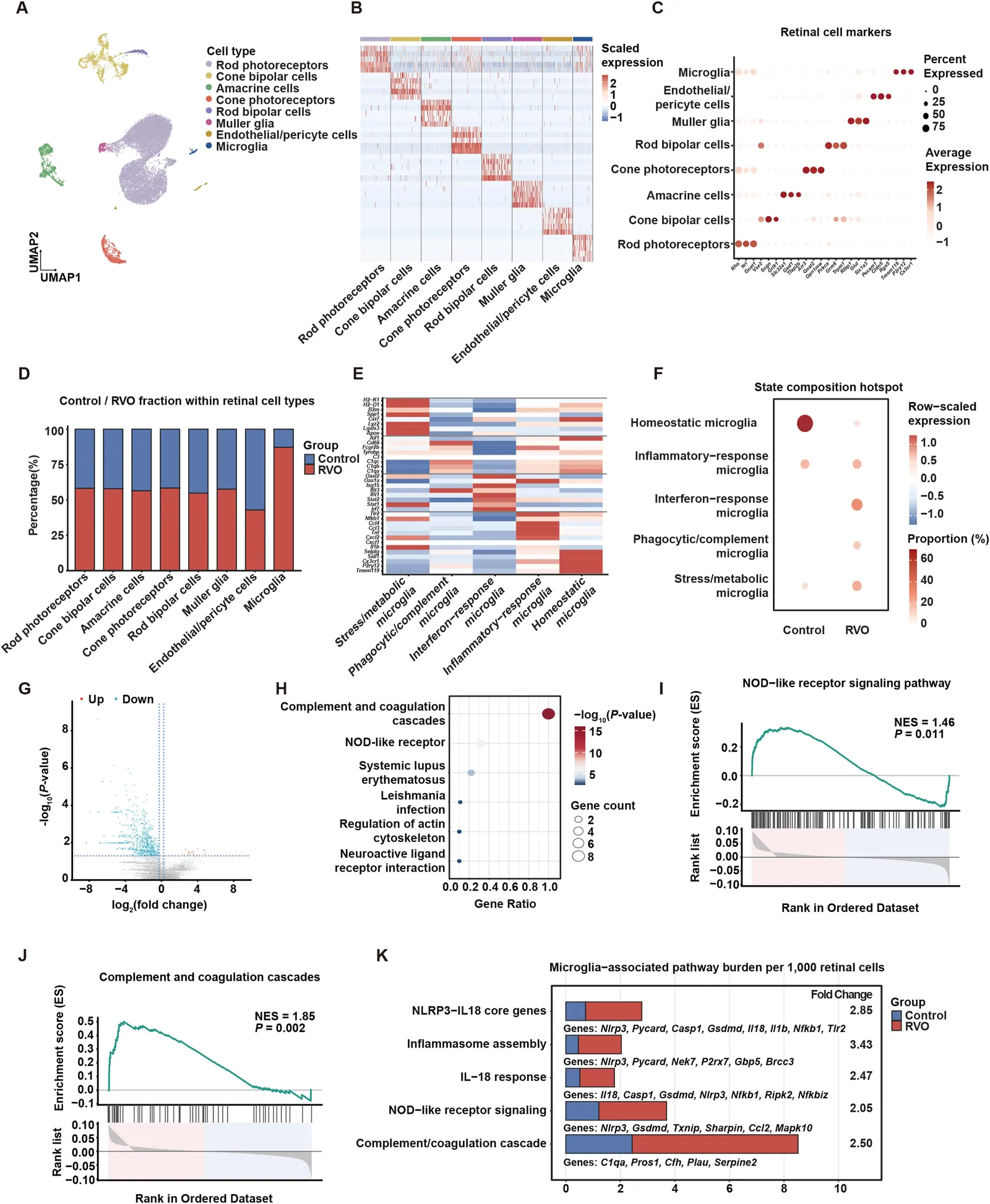
**Single-cell RNA sequencing identifies microglial inflammatory remodeling after RVO.** (A) Uniform manifold approximation and projection (UMAP) visualization of major retinal cell populations after quality-control filtering, comprising 6616 Control cells and 9134 RVO cells. (B, C) Heatmap and dot plot of canonical retinal cell markers used for cell-type annotation. In C, point size represents the percentage of cells expressing each marker, and color represents average expression. (D) Proportions of Control- and RVO-derived cells within each annotated retinal cell type. (E) Heatmap of marker-gene expression across homeostatic, inflammatory-response, interferon-response, phagocytic/complement, and stress/metabolic microglial states. Colors indicate row-scaled expression. (F) Functional-state composition of microglia from Control and RVO retinas. Point size and color intensity indicate the percentage of microglia assigned to each functional state within each group. (G) Volcano plot of microglial gene-expression differences between RVO and Control. Unadjusted *P* values were calculated using the Wilcoxon rank-sum test. Genes meeting nominal *P* < 0.05 and |log_2_ fold change| ≥ 0.25 are shown in red for increased expression and blue for decreased expression in RVO. These transcriptomic comparisons were exploratory because each condition was represented by one pooled library. (H) Pathway enrichment analysis of selected microglial genes. Point size represents gene count, and color represents −log_10_(*P*-value), based on unadjusted enrichment *P* values. (I, J) Gene set enrichment analysis of NOD-like receptor signaling and complement and coagulation cascades, respectively. Normalized enrichment scores (NES) and *P* values are shown. (K) Microglia-associated pathway burden normalized per 1000 retinal cells for the NLRP3–IL-18 core-gene, inflammasome-assembly, IL-18-response, NOD-like receptor signaling, and complement/coagulation programs. The genes included in each program and the corresponding fold changes are indicated. Each group comprised one pooled single-cell library prepared from 12 retinas from six animals.

Functional-state and differential-expression analyses further showed that RVO microglia shifted away from a homeostatic profile toward inflammatory, interferon-response, phagocytic/complement, and stress-associated programs (Fig. 3E–G and Fig. S1F). Exploratory pathway analysis of candidate microglial expression changes highlighted complement and coagulation cascades and NOD-like receptor signaling as the leading enriched pathways (Fig. 3H). GSEA indicated enrichment of NOD-like receptor signaling (NES = 1.46, *P* = 0.011) and complement/coagulation cascades (NES = 1.85, *P* = 0.002) in RVO-associated microglia (Fig. 3I and J). A microglia-associated pathway-burden analysis, normalized per 1000 retinal cells, showed that RVO increased the NLRP3–IL-18 core-gene burden by 2.85-fold, inflammasome-assembly burden by 3.43-fold, IL-18-response burden by 2.47-fold, NOD-like receptor burden by 2.05-fold, and complement/coagulation burden by 2.50-fold (Fig. 3K). These single-cell data identify retinal microglia as a prominent cellular compartment exhibiting inflammasome-linked transcriptional changes after RVO.

### 2.4 RVO induces microglial activation and NLRP3 inflammasome signaling

Retinas were collected 48 h after RVO induction for flow cytometry, quantitative reverse-transcription PCR (qRT-PCR), immunofluorescence, and western blotting to validate the transcriptomic findings at the cellular and molecular levels (Fig. 4A). Flow cytometry showed a higher proportion of resident microglia, defined as CD45^int^CD11b^+^ cells, in RVO retinas compared with sham retinas, using a gating strategy that excluded debris, doublets, and dead cells (Fig. 4B, C and Fig. S2). Retinal flat-mount imaging revealed a marked microglial response at the levels of both the superficial vascular plexus and the deep capillary plexus. In sham retinas, IBA1^+^ cells displayed a ramified morphology and sparse perivascular distribution, whereas RVO retinas contained denser IBA1^+^ cells with enlarged somata and shortened processes (Fig. 4D and E). Quantification showed that IBA1^+^ area increased from approximately 20% of the field of view in sham retinas to about 35–37% after RVO, while endpoint number and branch length fell to roughly one-half and three-fifths of sham values, respectively (Fig. 4F–H).

**Fig. 4:**
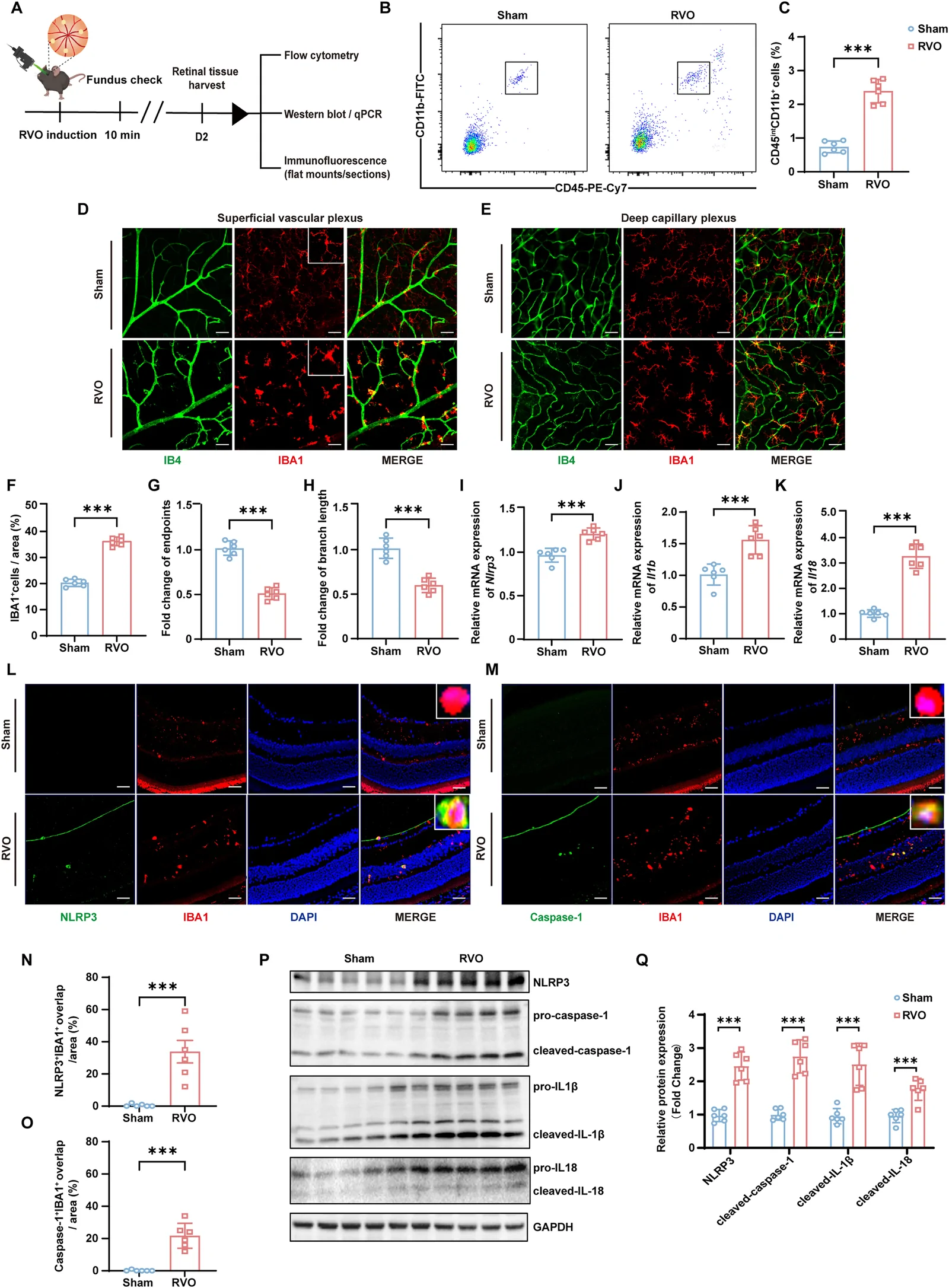
**RVO induces microglial activation and NLRP3 inflammasome engagement.** (A) Schematic of RVO induction and retinal tissue collection on day 2 (D2) for flow cytometry, western blotting, quantitative reverse-transcription PCR (qRT-PCR), and immunofluorescence. (B, C) Representative flow-cytometry plots and quantification of CD45^int^CD11b^+^ retinal microglia in sham and RVO retinas. (D, E) Retinal flat-mount immunofluorescence of isolectin GS-IB4-labeled vasculature and IBA1^+^ microglia at the superficial vascular plexus and deep capillary plexus. (F–H) Quantification of IBA1^+^ area, microglial endpoint number, and branch length, respectively. (I–K) qRT-PCR analysis of *Nlrp3*, *Il1b*, and *Il1*8 mRNA expression in retinal tissue. (L, M) Retinal-section immunofluorescence showing colocalization of NLRP3 and caspase-1, respectively, with IBA1^+^ microglia. Insets show representative areas of colocalization at higher magnification. (N, O) Quantification of NLRP3–IBA1 and caspase-1–IBA1 colocalized areas, respectively. (P) Western blot analysis of NLRP3 and the pro- and cleaved forms of caspase-1, IL-1β, and IL-18. (Q) Densitometric quantification of NLRP3, cleaved caspase-1, cleaved IL-1β, and cleaved IL-18. Scale bars, 50 μm. Data in C, F–K, N, O, and Q are presented as the mean ± SD; *n* = 6 animals per group. Comparisons were performed using Welch's unpaired *t*-test. ***P < 0.001.

Inflammasome-related changes accompanied the microglial response. qRT-PCR showed increased retinal expression of *Nlrp3*, *Il1b*, and *Il18* after RVO, with *Il18* exhibiting the largest increase, at approximately threefold relative to sham retinas (Fig. 4I–K). Immunofluorescence of retinal sections demonstrated increased colocalization of NLRP3 and caspase-1 with IBA1^+^ microglia after RVO, with quantitative analysis confirming larger NLRP3–IBA1 and caspase-1–IBA1 colocalized areas (Fig. 4L–O). Western blotting further demonstrated increased retinal levels of NLRP3, cleaved caspase-1, cleaved IL-1β, and cleaved IL-18 after RVO, consistent with activation of the NLRP3 inflammasome (Fig. 4P and Q).

### 2.5 Genetic disruption of *Nlrp3* or *Il18* attenuates RVO-induced neuroinflammation and retinal injury

To determine whether NLRP3 contributes functionally to RVO pathology, mice with constitutive global deletion of *Nlrp3* (*Nlrp3*^−/−^) and wild-type (WT) controls were subjected to experimental RVO, with deletion confirmed by genotyping (Fig. S3A). IR fundus imaging, FFA, and OCT were performed on D1, followed by ERG on D2, and retinas from separate terminal cohorts were collected on D2 for molecular and cellular analyses (Fig. 5A). Compared with WT-RVO eyes, *Nlrp3*^−/−^-RVO eyes showed less vascular leakage, retinal whitening, and OCT-defined retinal thickening (Fig. 5B). Whole retina thickness increased to approximately 340–350 μm in WT-RVO eyes but was limited to approximately 270–290 μm in *Nlrp3*^−/−^-RVO eyes. Attenuated thickening was also observed in the inner retina, INL, and NFL–GCL (Fig. 5C–F). ERG analysis showed partial preservation of retinal electrophysiological function in *Nlrp3*^−/−^-RVO eyes compared with WT-RVO eyes, as reflected by higher b-wave amplitudes, b/a wave ratios, and total OP amplitudes (Fig. 5G–J).

**Fig. 5:**
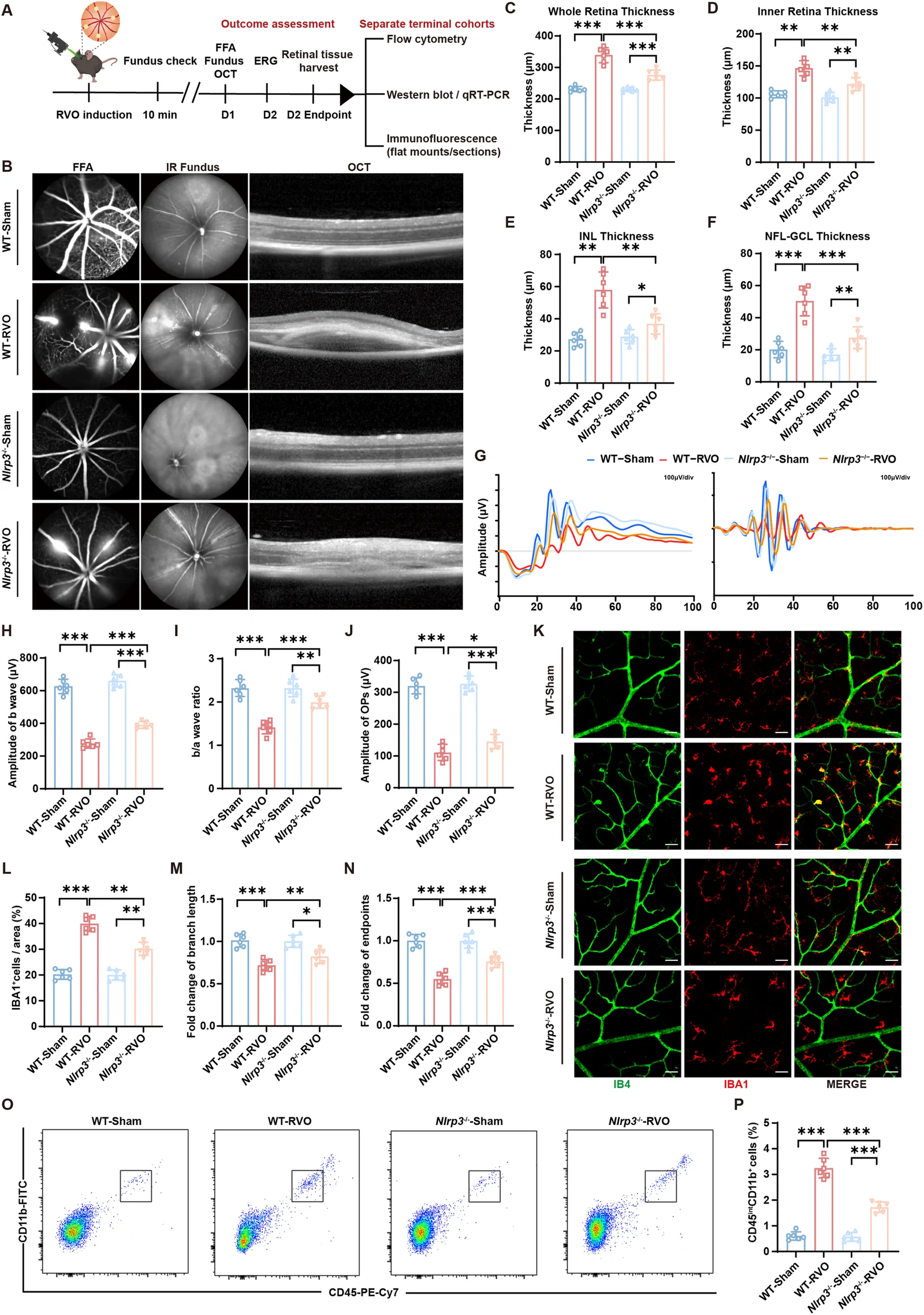
**Constitutive global deletion of *Nlrp3* attenuates retinal thickening, electrophysiological impairment, and microglial activation after RVO.** (A) Schematic of RVO induction and outcome assessment in WT and *Nlrp3*^−/−^ mice. FFA, IR fundus imaging, and OCT were performed on day 1 (D1), and ERG was performed on day 2 (D2). Separate terminal cohorts were collected on D2 for flow cytometry, western blotting, qRT-PCR, and immunofluorescence. (B) Representative FFA, IR fundus, and OCT images from WT-sham, WT-RVO, *Nlrp3*^−/−^-sham, and *Nlrp3*^−/−^-RVO eyes. (C–F) Quantification of whole retinal, inner-retinal, INL, and NFL–GCL thickness, respectively. (G) Representative scotopic ERG and OP traces. (H–J) Quantification of b wave amplitude, b/a wave ratio, and total OP amplitude, respectively, from responses elicited at 3.0 cd s/m^2^. (K) Retinal flat-mount immunofluorescence of isolectin GS-IB4-labeled vasculature and IBA1^+^ microglia in the deep capillary plexus. (L–N) Quantification of IBA1^+^ area, microglial branch length, and endpoint number, respectively. (O, P) Representative flow-cytometry plots and quantification of CD45^int^CD11b^+^ retinal microglia. Scale bars in K, 50 μm. Data in C–F, H–J, L–N, and P are presented as the mean ± SD; *n* = 6 animals per group. Comparisons were performed using two-way ANOVA followed by Šídák's multiple-comparisons test. *P < 0.05, **P < 0.01, and ***P < 0.001. ERG, electroretinography; FFA, fundus fluorescein angiography; INL, inner nuclear layer; IR, infrared reflectance; NFL–GCL, nerve fiber layer–ganglion cell layer; OCT, optical coherence tomography; OP, oscillatory potential; qRT-PCR, quantitative reverse-transcription PCR; WT, wild-type.

*Nlrp3* deletion also attenuated the microglial response to RVO*.* Retinal flat-mount imaging showed a smaller IBA1^+^ area and partial restoration of branch-length and endpoint indices in *Nlrp3*^−/−^-RVO retinas compared with WT-RVO retinas (Fig. 5K–N). Flow cytometry likewise showed a lower proportion of CD45^int^CD11b ^+^ cells in *Nlrp3*^−/−^-RVO retinas (Fig. 5O and P). Consistently, *Nlrp3*^−/−^-RVO retinas showed lower *Il1b* and *Il18* transcript levels, reduced cleaved caspase-1, cleaved IL-1β, and cleaved IL-18 on western blotting, and weaker microglia-associated NLRP3/caspase-1 signals (Fig. S3B–L).

We further examined IL-18 as a downstream effector. Constitutive global *Il18*^−/−^ mice showed a similar protective phenotype after RVO. Compared with WT-RVO eyes, *Il18*^−/−^-RVO eyes had reduced retinal thickening, better preservation of ERG responses, and attenuated microglial activation after RVO (Fig. S4A–P and Fig. S5). Constitutive global deletion of either gene attenuated acute structural, electrophysiological, and inflammatory abnormalities after RVO, supporting functional involvement of NLRP3–IL-18 signaling at the whole-animal level.

### 2.6 MG1-MCC950 nanoliposomes exhibit uniform nanoscale properties, colloidal stability, and gradual drug release

Guided by the microglial NLRP3–IL-18 axis, we engineered MG1-functionalized, MCC950-loaded nanoliposomes for microglia-targeted intraocular delivery (Fig. 6A). MG1, a nine-amino-acid peptide with the sequence CHHSSSARC, was conjugated to DSPE-PEG-NHS to generate DSPE-PEG-MG1, and product formation was supported by the corresponding ^1^H NMR spectra (Fig. S6). The nanoliposomal platform comprised SPC/cholesterol liposomes loaded with MCC950 and surface-functionalized with DSPE-PEG-MG1. DSPE-PEG-Cy5 was incorporated into fluorescently labeled formulations for subsequent tracking. Transmission electron microscopy (TEM) showed spherical or near-spherical particles without obvious aggregation, while dynamic light scattering (DLS) revealed unimodal nanoscale size distributions across the tested formulations (Fig. 6B and C). The mean hydrodynamic diameter of MG1-MCC950 NPs was 138.50 ± 2.05 nm (Fig. 6D). All formulations exhibited negative zeta potentials, with a value of −24.19 ± 2.08 mV for MG1-MCC950 NPs (Fig. 6E). Additional physicochemical parameters are summarized in Table S1.

**Fig. 6:**
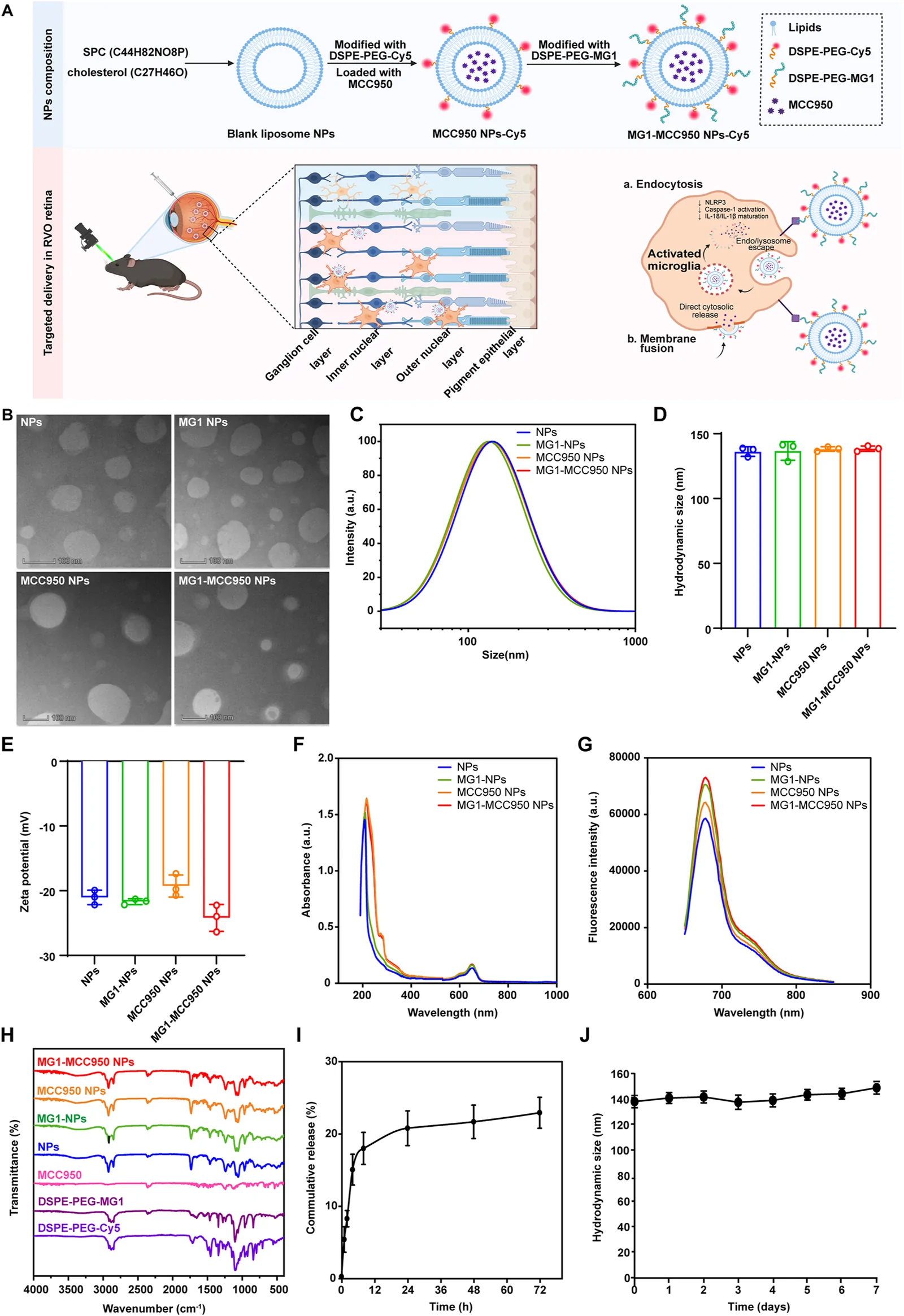
**Construction and physicochemical characterization of MG1-functionalized MCC950-loaded nanoliposomes.** (A) Schematic illustration of nanoliposome composition and microglia-targeted delivery in RVO, showing the SPC/cholesterol lipid bilayer, MCC950 payload, DSPE-PEG-Cy5 labeling, and DSPE-PEG-MG1 functionalization. Proposed cellular uptake via endocytosis or membrane fusion and subsequent intracellular drug release are illustrated. (B) Representative TEM images of blank NPs, MG1-NPs, MCC950 NPs, and MG1-MCC950 NPs. Scale bars, 100 nm. (C) DLS size-distribution profiles of the indicated formulations. (D) Hydrodynamic diameters. (E) Zeta potentials. (F) UV–Vis absorption spectra. (G) Cy5 fluorescence emission spectra. (H) FTIR spectra of the individual components and nanoliposomal formulations. (I) *In vitro* cumulative release profile of MCC950 from MG1-MCC950 NPs at 37 °C. (J) Hydrodynamic diameter of MG1-MCC950 NPs during incubation in PBS at 37 °C for 7 days. Data in D, E, I, and J are presented as the mean ± SD of three independently prepared batches per formulation (*n* = 3). Technical triplicate measurements were averaged within each batch before calculating the mean and SD across batches. Representative images, size-distribution profiles, and spectra are shown in B, C, and F–H, respectively. DLS, dynamic light scattering; FTIR, Fourier-transform infrared spectroscopy; NPs, nanoparticles; PBS, phosphate-buffered saline; SD, standard deviation; SPC, soybean phosphatidylcholine; TEM, transmission electron microscopy; UV–Vis, ultraviolet–visible.

UV–visible spectroscopy showed a Cy5-associated absorption band near 650 nm in Cy5-containing formulations, while MCC950-loaded formulations exhibited an additional UV-region feature near 286 nm (Fig. 6F). Following excitation at 620 nm, Cy5-containing nanoparticles displayed an emission maximum near 678 nm (Fig. 6G). Fourier-transform infrared spectroscopy (FTIR) showed that the formulated nanoparticles retained spectral features attributable to their lipid, MCC950, DSPE-PEG-Cy5, and DSPE-PEG-MG1 components, with modest peak shifts consistent with their incorporation into the liposomal system (Fig. 6H). *In vitro* release followed a biphasic profile, with an initial release phase followed by slower release, reaching approximately 23% cumulative MCC950 release at 72 h (Fig. 6I). The mean hydrodynamic diameter changed only modestly during incubation in PBS at 37 °C for 7 days, with no marked increase indicative of particle aggregation (Fig. 6J). These measurements demonstrate that MG1 functionalization and MCC950 loading yielded negatively charged nanoscale liposomes with short-term colloidal stability and gradual MCC950 release *in vitro*.

### 2.7 MG1 functionalization promotes preferential nanoliposome association with activated microglia *in vitro* and retinal microglia *in vivo*

We next evaluated whether MG1 modification improves microglial targeting. In BV2 cells, lipopolysaccharide (LPS) stimulation increased NLRP3 and CD86 expression, confirming induction of an inflammatory phenotype (Fig. S7A, D and F). Flow cytometry showed a marked rightward shift in Cy5 fluorescence in LPS-stimulated BV2 cells exposed to MG1-NPs compared with those exposed to non-targeted NPs, with the mean cell-associated fluorescence increasing from approximately 250 to more than 600 a.u. (Fig. S7B and C). Confocal imaging similarly showed greater MG1-NPs-associated fluorescence in LPS-stimulated BV2 cells than in unstimulated cells (Fig. S7D and E).

MG1 functionalization also promoted microglia-enriched nanoparticle localization *in vivo*. Following intravitreal administration in RVO eyes, flow-cytometric analysis of CD45^int^CD11b^+^ retinal microglia showed a pronounced rightward shift in Cy5 fluorescence and an approximately 2.5-fold higher mean fluorescence intensity with MG1-NPs than with non-targeted NPs (Fig. S7G and H). Retinal imaging demonstrated increased colocalization of MG1-NPs-associated fluorescence with IBA1^+^ microglia, while the proportion of NPs-positive microglia increased from approximately 15% with non-targeted NPs to approximately 75% with MG1-NPs (Fig. S7I and J).

We next examined the cellular distribution of MG1-NPs within the RVO retina. Flow cytometric analysis distinguished CD45^int^CD11b^+^ microglia from CD45^high^CD11b^+^ infiltrating myeloid cells and showed significantly greater MG1-NPs-associated fluorescence in microglia (Fig. S8A–C). Confocal and orthogonal imaging showed prominent MG1-NPs localization in IBA1^+^ cells but substantially lower localization in GFAP^+^ glial cells and RBPMS^+^ retinal ganglion cells (RGCs) (Fig. S8D–F). Detectable MG1-NPs fluorescence was observed in approximately 75% of IBA1^+^ cells, compared with approximately 4–6% of GFAP^+^ glial cells and RBPMS^+^ retinal ganglion cells (Fig. S8F). These findings show that MG1 functionalization therefore increased nanoliposome association with activated BV2 cells and promoted preferential localization to retinal microglia after RVO.

### 2.8 MG1-MCC950 nanoliposomes attenuate retinal thickening, electrophysiological impairment, and microglial activation after RVO

Before comparing the therapeutic formulations, we performed an initial *in vivo* dose-ranging experiment using intravitreal MCC950. Immunoblotting showed reductions in NLRP3, cleaved caspase-1, cleaved IL-1β, and cleaved IL-18 across the tested concentration range of 0.01–10 mM. Based on the overall inflammasome-inhibition profile, 1 mM MCC950 was selected for subsequent therapeutic experiments (Fig. S9A). At an injection volume of 1.0 μL, this concentration corresponded to an administered MCC950 dose of 1 nmol per eye. Six hours after RVO induction, eyes received a single intravitreal injection of PBS, free MCC950, non-targeted MCC950 NPs, or MG1-MCC950 NPs. All active formulations delivered an equivalent MCC950 dose of 1 nmol per eye, whereas the PBS group received 1.0 μL of PBS without MCC950. FFA, IR fundus imaging, and OCT were performed on D1, followed by ERG and terminal tissue collection from separate cohorts on D2 (Fig. 7A).

**Fig. 7:**
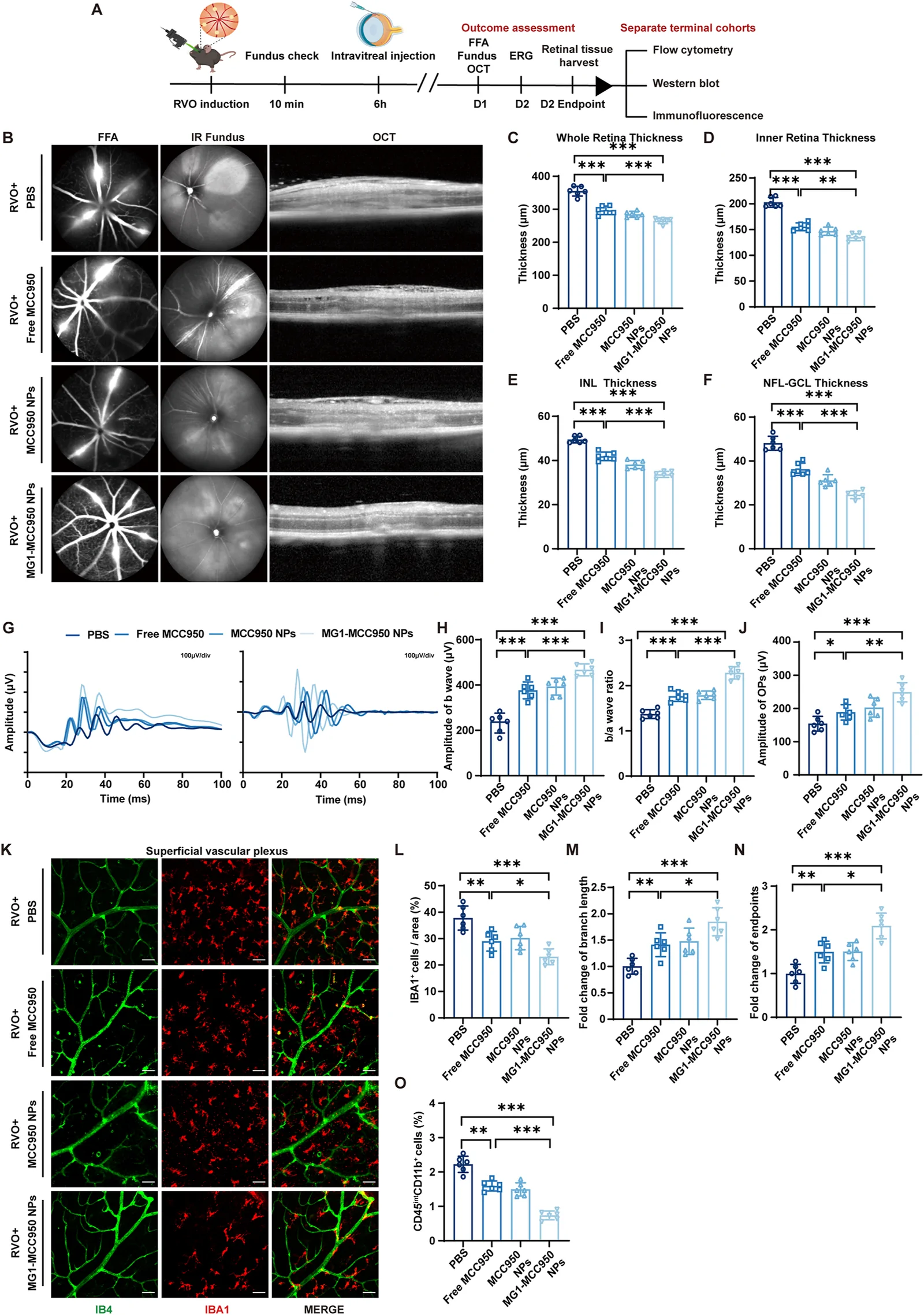
**MG1-MCC950 nanoliposomes attenuate retinal thickening, electrophysiological impairment, and microglial activation after RVO.** (A) Experimental schedule for the therapeutic study. A single 1.0-μL intravitreal injection was administered 6 h after RVO induction. Free MCC950, MCC950 NPs, and MG1-MCC950 NPs each delivered an MCC950-equivalent dose of 1 nmol per eye; PBS served as the vehicle control. Retinal imaging was performed on day 1 (D1), and ERG was performed on day 2 (D2). Separate terminal cohorts were euthanized on D2 for cellular and molecular analyses. (B) Representative FFA, IR fundus, and OCT images from RVO eyes treated with PBS, free MCC950, MCC950 NPs, or MG1-MCC950 NPs. (C–F) Quantification of whole retinal, inner retinal, inner nuclear layer (INL), and nerve fiber layer–ganglion cell layer (NFL–GCL) thickness, respectively. (G) Representative scotopic ERG and OP waveforms. (H–J) Quantification of b wave amplitude, b/a wave ratio, and total OP amplitude, respectively, from responses elicited at 3.0 cd s/m^2^. (K) Retinal flat-mount immunofluorescence of isolectin GS-IB4-labeled vasculature and IBA1^+^ microglia in the superficial vascular plexus. (L–N) Quantification of IBA1^+^ area and fold changes in microglial branch length and endpoint number, respectively. (O) Flow-cytometric quantification of CD45^int^CD11b^+^ retinal microglia. Scale bars in K, 50 μm. Data in C–F, H–J, and L–O are presented as the mean ± SD; *n* = 6 animals per group. Statistical comparisons were performed using one-way ANOVA followed by Tukey's multiple-comparisons test. *P < 0.05, **P < 0.01, and ***P < 0.001. ERG, electroretinography; FFA, fundus fluorescein angiography; IR, infrared reflectance; NPs, nanoparticles; OCT, optical coherence tomography; OP, oscillatory potential; PBS, phosphate-buffered saline; RVO, retinal vein occlusion.

Multimodal imaging showed the greatest structural protection in eyes treated with MG1-MCC950 NPs. PBS-treated RVO eyes exhibited pronounced vascular leakage, retinal whitening, and retinal thickening. In contrast, eyes treated with MG1-MCC950 NPs showed less vascular leakage and whitening, together with better-preserved retinal architecture, than those treated with free MCC950 or non-targeted MCC950 NPs (Fig. 7B). Whole retinal thickness was approximately 350 μm in PBS-treated RVO eyes but only 265–270 μm in eyes treated with MG1-MCC950 NPs. Corresponding reductions were observed in inner-retinal, INL, and NFL–GCL thickness (Fig. 7C–F). ERG recordings further demonstrated greater preservation of retinal electrophysiological function in the MG1-MCC950 NP group. The b-wave amplitude was 239.0 ± 43.6 μV in PBS-treated eyes compared with 467.0 ± 26.1 μV in eyes treated with MG1-MCC950 NPs. Treatment with MG1-MCC950 NPs also resulted in a higher b/a wave ratio and greater total OP amplitude (Fig. 7G–J).

Treatment with MG1-MCC950 NPs also attenuated the retinal microglial response. Retinal flat-mount imaging showed a smaller IBA1^+^ area and greater preservation of microglial branch length and endpoint number than treatment with free MCC950 or non-targeted MCC950 NPs (Fig. 7K–N). Flow cytometry showed that CD45^int^CD11b^+^ cells accounted for approximately 2.1–2.3% of the analyzed population in PBS-treated RVO retinas but less than 1% following treatment with MG1-MCC950 NPs (Fig. 7O and Fig. S10A). Additional imaging of the deep vascular plexus corroborated the reduction in IBA1 immunoreactivity (Fig. S10B). Western blotting further showed lower retinal levels of NLRP3, cleaved caspase-1, cleaved IL-1β, and cleaved IL-18 following treatment with MG1-MCC950 NPs than with free MCC950 or non-targeted MCC950 NPs (Fig. S10C–G). Collectively, these findings indicate that MG1 functionalization enhances the therapeutic efficacy of MCC950-loaded nanoliposomes after RVO, resulting in greater suppression of microglial activation and NLRP3–IL-18 signaling together with improved structural and electrophysiological outcomes.

### 2.9 Preliminary biosafety assessment of MG1-MCC950 nanoliposomes

A preliminary biosafety assessment showed no overt adverse effects of MG1-MCC950 NPs under the tested conditions. At 7 days after a single intravitreal administration, representative hematoxylin and eosin (H&E)-stained sections of the heart, liver, spleen, lung, and kidney showed comparable tissue architecture across treatment groups, without apparent treatment-associated histopathological abnormalities (Fig. S11A). *In vitro* CCK-8 assays further showed that exposure to free MCC950, MCC950 NPs, or MG1-MCC950 NPs at concentrations of 1, 10, or 100 μM for 24 or 48 h did not materially reduce the viability of BV2 or ARPE-19 cells, which remained near control levels across the tested conditions (Fig. S11B). These findings support the short-term systemic histological tolerability and *in vitro* cytocompatibility of MG1-MCC950 nanoliposomes under the conditions examined.

## 3 Discussion

Venous outflow disorders exhibit heterogeneous anatomical abnormalities and clinical manifestations, as illustrated by recent work on cerebral venous outflow insufficiency [28]. RVO is primarily classified as a venous occlusive disease, but retinal injury after venous obstruction cannot be fully explained by vascular leakage alone [29]. Increasing clinical evidence indicates that intraocular inflammatory mediators correlate with macular edema and retinal structural changes in RVO patients, suggesting that inflammation actively contributes to disease progression rather than merely reflecting tissue damage [30]. Our findings extend this clinical evidence by identifying microglia-associated NLRP3–IL-18 signaling as a prominent inflammatory feature of early experimental RVO. By integrating bulk and single-cell transcriptomics, spatial protein analysis, genetic loss-of-function models, and targeted nanotherapeutic intervention, this study connects an early inflammatory mechanism with a potential therapeutic strategy.

A key implication of these findings is that microglia serve as active effector cells linking vascular occlusion to secondary neuroretinal injury. Microglia are highly responsive to ischemic stress, extracellular danger signals, and barrier disruption, all of which are likely present after acute RVO. Once activated, microglia can release cytokines, complement-related mediators, and chemokines that reshape the retinal inflammatory microenvironment and further impair neurovascular homeostasis. This interpretation is consistent with experimental studies in retinal ischemia-reperfusion injury showing that modulation of microglial activation can reduce neuronal damage and preserve retinal electrophysiological function [31], [32]. Although RVO and ischemia-reperfusion injury are distinct models, both support the broader concept that microglia-mediated inflammation is a modifiable driver of ischemic retinal damage.

The NLRP3 inflammasome provides a plausible molecular link between ischemic stress and inflammatory amplification. NLRP3 activation requires priming signals, often involving NF-κB-dependent induction of inflammasome components, as well as activation signals such as ionic imbalance, mitochondrial dysfunction, oxidative stress, and lysosomal stress. These upstream events are highly compatible with the hypoxic and edematous retinal environment after venous occlusion. In the present study, constitutive global deletion of *Nlrp3* attenuated retinal thickening, microglial activation, inflammasome signaling, and retinal electrophysiological impairment after RVO. Global *Il18* deletion produced a similar protective phenotype, supporting IL-18 as a functionally relevant component of the downstream inflammatory response. In conjunction with the prominent localization of NLRP3 and caspase-1 immunoreactivity to IBA1^+^ cells, these findings support a model in which microglia-associated NLRP3–IL-18 signaling amplifies acute retinal injury after RVO. This pathway may also participate in a feed-forward inflammatory circuit that reinforces microglial activation and prolongs disruption of retinal homeostasis.

The therapeutic effects of MCC950 further support the relevance of NLRP3 inhibition in ischemic and metabolically stressed retinal tissue. In an oxygen-induced retinopathy model, MCC950 attenuated retinal apoptosis and improved impaired autophagic flux through regulation of the AMPK/mTOR/ULK1 pathway [33], and a recent study of diabetic retinopathy similarly reported that intravitreal MCC950 suppressed ROS–NEK7–NLRP3 signaling, with 1 mM producing marked retinal protection in that experimental setting [34]. Although these models differ from RVO, they provide independent evidence that NLRP3 inhibition can modulate inflammatory, oxidative, and cell-death pathways across several forms of retinal stress. In our RVO model, dose screening identified 1 mM MCC950 as an effective concentration for suppressing inflammasome-related proteins without using the highest concentration tested. Moreover, a single intravitreal administration of MG1-MCC950 nanoliposomes at 6 h after RVO induction produced greater suppression of NLRP3 inflammasome signaling and greater structural and electrophysiological protection than free MCC950 or non-targeted MCC950 nanoliposomes. The convergence of genetic and pharmacological findings therefore supports NLRP3–IL-18 signaling as a therapeutically actionable component of acute RVO injury.

This strategy also highlights a general principle for treating ischemic retinal diseases: anti-inflammatory therapy may be more effective when matched to the dominant pathogenic cell population. Current RVO management largely focuses on vascular leakage, especially through anti-VEGF therapy, but some patients continue to experience recurrent edema, incomplete functional recovery, or progressive retinal damage. Microglia-targeted inflammasome inhibition could therefore complement vascular-directed approaches by addressing the inflammatory component of RVO. From a translational perspective, lipid-based nanocarriers are attractive for intraocular delivery because they can improve drug retention, support sustained release, and permit surface modification for cellular targeting [35]. The gradual release, short-term colloidal stability, and preliminary biocompatibility observed here provide an initial foundation for further development of this formulation.

Several limitations should be considered. First, the focus on acute experimental RVO and the absence of vessel-level flow classification limited assessment of long-term reperfusion and genotype-dependent vascular responses. OCT measurements adjacent to photocoagulation sites may also reflect local laser effects. Second, retinal pooling yielded one library per condition in each sequencing experiment, preventing estimation of inter-animal variability. The transcriptomic analyses therefore served to generate hypotheses, with the selected NLRP3–IL-18 pathway subsequently evaluated in independent animal cohorts. Third, constitutive global deletion of *Nlrp3* and *Il18* does not distinguish the contributions of microglia from those of other retinal or infiltrating immune cells, nor exclude systemic or developmental effects. Finally, MG1 targeting was assessed primarily using nanoparticle-associated fluorescence. The formulation's pharmacokinetics, intracellular trafficking, long-term efficacy, and safety require further evaluation, including studies of delayed or repeated dosing and studies in larger-animal models.

## 4 Conclusions

This study identifies microglia-associated NLRP3–IL-18 signaling as a critical inflammatory amplifier in early experimental RVO and demonstrates that global deletion of either *Nlrp3* or *Il18* attenuates acute retinal injury, microglial activation, and electrophysiological dysfunction. MG1 functionalization promoted preferential nanoliposome association with activated retinal microglia and achieved greater inflammasome suppression and retinal protection than free MCC950 or non-targeted MCC950-loaded nanoliposomes. Collectively, these findings support further investigation of microglia-targeted inflammasome inhibition as a complementary, mechanism-based therapeutic strategy for RVO and other ischemic retinal diseases (Fig. 8).

**Fig. 8:**
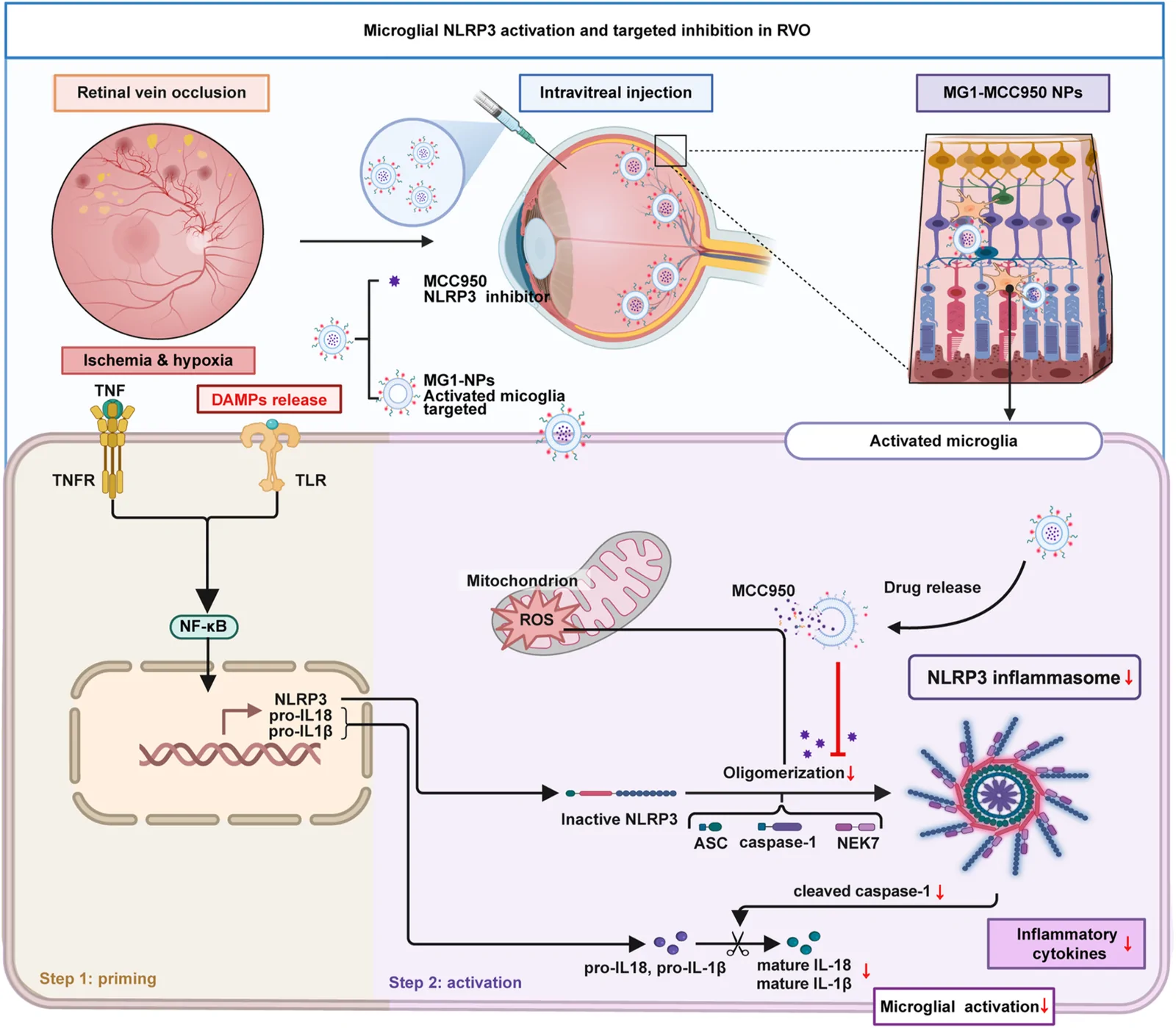
**Working model of preferential inflammasome inhibition by MG1-MCC950 nanoliposomes after retinal vein occlusion.** Retinal venous occlusion induces ischemic stress and danger-associated signaling, which promote TLR/NF-κB priming, microglial activation, NLRP3 inflammasome assembly, caspase-1 cleavage, and maturation of IL-1β and IL-18. MG1-modified MCC950-loaded nanoliposomes preferentially interact with activated microglia, enhance intracellular MCC950 delivery, inhibit NLRP3 inflammasome signaling, and reduce retinal edema, inflammatory activation, and functional impairment.

## 5 Methods

### 5.1 Animals and anesthesia

All animal procedures complied with the ARVO Statement and were approved by the Institutional Animal Care and Use Committee of Capital Medical University (AEEI-2026-139). Age-matched C57BL/6J mice, *Nlrp3* knockout mice (*Nlrp3*^−/−^), *Il18* knockout mice (*Il18*^−/−^), and corresponding wild-type (WT) littermate controls on a C57BL/6J background were used as indicated. Mice of both sexes were randomly assigned when required and housed under a 12 h light/dark cycle with free access to food and water. Investigators were blinded to group allocation during imaging and quantitative analyses whenever feasible. General anesthesia was induced by intraperitoneal injection of 1% sodium pentobarbital (0.15 mL/20 g body weight; 75 mg/kg). Adequate anesthetic depth was confirmed by the absence of pedal withdrawal reflex. Pupils were dilated before ocular procedures, and topical ocular anesthesia was applied when appropriate.

### 5.2 Establishment of RVO model

Experimental RVO was induced by Rose Bengal-assisted laser photothrombosis as previously described with minor modifications [18]. Rose Bengal (40 mg/kg; 8 mg/mL) was administered via the tail vein. After a 10-min circulation period, the retinal vasculature was visualized using a corneal cover glass and carbomer gel. Three or four major retinal veins located 2–3 optic-disc diameters from the optic-disc margin were targeted in each eye using a 532-nm laser with a 50-μm spot diameter, 1-s exposure duration, and 50-mW power. Four or five pulses were delivered to each targeted vein until visible venous narrowing, congestion, or interruption of the blood column was observed.

Approximately 10 min after irradiation, each treated eye underwent direct funduscopic reassessment according to prespecified procedural eligibility criteria. Eligible eyes showed funduscopic evidence of impaired venous flow at the treated sites. In three-vein protocols, all three treated veins were required to meet the eligibility criteria, whereas in four-vein protocols, at least three of the four treated veins were required to meet these criteria. Eyes were not carried forward if the required signs were absent or were lost early, including early restoration of visible blood-column continuity, or if anesthesia-related complications, hypothermia, persistent ocular media opacity, extensive preretinal hemorrhage, accidental injury to an adjacent retinal artery, or another condition precluded reliable downstream assessment.

Sham eyes received the same Rose Bengal administration and procedural handling, with matched laser exposure applied between major retinal vessels at the corresponding distance from the optic disc.

### 5.3 Intravitreal injection

Injection volume was controlled using a 2.5-μL Hamilton 62 RN precision microsyringe (600 Series, PN 7632-01; Hamilton Company, Reno, NV, USA) fitted with a removable 33-gauge needle. Before each injection, air bubbles were expelled and the injection volume was set to 1.0 μL using the calibrated barrel graduations. Intravitreal injections were performed under a surgical microscope 6 h after RVO induction. A small entry site was created approximately 1.0 mm posterior to the limbus, and the needle was advanced obliquely into the mid-vitreous cavity, with the tip maintained posterior to the lens and clear of the retina and major retinal vessels. The solution was delivered slowly over approximately 10 s. The needle was then held in place for an additional 10 s before being withdrawn slowly to minimize reflux.

Free MCC950, MCC950 NPs, and MG1-MCC950 NPs were administered at an MCC950-equivalent concentration of 1 mM in a volume of 1.0 μL, corresponding to 1 nmol MCC950 per eye. PBS (1.0 μL) served as the vehicle control. The concentrations of MCC950 NPs and MG1-MCC950 NPs were adjusted according to the measured MCC950 content of the purified formulations after removal of free, unencapsulated drug, rather than the amount of MCC950 initially added during nanoparticle preparation.

Technical success required appropriate intravitreal positioning of the needle tip, delivery of the full calibrated volume, absence of visible reflux, and preservation of ocular integrity after needle withdrawal. Eyes exhibiting evident reflux, lens injury, vitreous or retinal hemorrhage, retinal penetration, or retinal detachment were considered technically unsuccessful and excluded from subsequent analyses. These criteria were applied before experimental outcomes were assessed.

### 5.4 Preparation of MG1/Cy5-modified MCC950-loaded nanoliposomes

MG1, a nine-amino-acid peptide with the sequence CHHSSSARC, was purchased from Ruixitech (Xi'an, China). The PEG component had an average molecular weight of 2 kDa. DSPE-PEG-MG1 was synthesized via an amide-coupling reaction between DSPE-PEG-NHS and the N-terminal amino group of MG1. Briefly, DSPE-PEG-NHS (100 mg) was dissolved in 3 mL of *N,N*-dimethylformamide (DMF), followed by the addition of MG1 at 1.1 molar equivalents and triethylamine at 3.0 molar equivalents relative to DSPE-PEG-NHS. The reaction was allowed to proceed at room temperature for 12 h. The reaction mixture was then transferred to dialysis tubing with a molecular-weight cutoff of 1000 Da and dialyzed against purified water for 24 h to remove residual low-molecular-weight reactants and other impurities. The retained product was collected and lyophilized to obtain DSPE-PEG-MG1. Product formation was evaluated by comparing the ^1^H NMR spectra of MG1 and DSPE-PEG-MG1, recorded at 400 MHz in DMSO-*d*_6_.

MCC950 sodium (MedChemExpress, Cat. No. HY-12815A), hereafter referred to as MCC950, was used throughout the study. MG1/Cy5-modified MCC950-loaded liposomal nanoparticles were prepared by thin-film hydration. Briefly, soybean phosphatidylcholine (SPC, 90 mg), cholesterol (3 mg), DSPE-PEG-Cy5 (2 mg), DSPE-PEG-MG1 (5 mg), and MCC950 (8 mg) were dissolved in chloroform (5 mL). The organic solvent was evaporated under reduced pressure to form a uniform lipid film, which was subsequently hydrated with deionized water (5 mL). The suspension was sonicated and extruded through a 100 nm polycarbonate membrane to obtain size-controlled nanoparticles. Free MCC950 and unincorporated components were removed by nanodialysis using a 30 nm polycarbonate membrane for 3 h. Following dialysis, the dispersion was brought to a final volume of 10 mL with deionized water. Non-targeted MCC950-loaded liposomes and blank liposomes with or without MG1/Cy5 modification were prepared using the same procedure with the corresponding lipid compositions.

### 5.5 Physicochemical characterization of nanoliposomes

The particle size and zeta potential of the prepared nanoparticles were measured using a particle size and zeta potential analyzer (NanoBrook 90Plus PALS, Brookhaven). The nanoparticle morphology was visualized via TEM (TF20, FEI) after negative staining. The drug loading (DL) and encapsulation efficiency (EE) of MCC950 were determined by first measuring the absorbance after treatment with Triton X-100 using an ultraviolet spectrophotometer, followed by comparison with the MCC950 concentration-absorbance standard curve. DL was calculated as the mass of MCC950 encapsulated in the nanoparticles divided by the total mass of the nanoparticles, and EE was calculated as the mass of encapsulated MCC950 divided by the initial mass of MCC950 added.

The *in vitro* profile of MCC950 release from MG1-MCC950 NPs was evaluated as follows. First, MG1-MCC950 NPs were dispersed in release medium, the dispersion was placed in a dialysis device (pore size: 30 nm), and the dialysis device was immersed in release medium with gentle stirring at 37 °C. Samples were removed at the indicated times (0, 1, 2, 4, 8, 12, 24, 48, and 72 h) for MCC950 concentration determination.

The full ultraviolet (UV) absorption spectra of the samples were measured from 200 to 1000 nm using a UV–Vis absorption spectrometer. The fluorescence emission spectra were determined using a multifunctional microplate reader to verify the Cy5 fluorescence signal. Three independent batches of each formulation were prepared. Quantitative physicochemical measurements were performed in triplicate for each batch, and the results are presented as the mean ± SD of the three batch means (*n* = 3).

### 5.6 Cell culture and *in vitro* assays

BV2 microglial cells and ARPE-19 cells were used for nanoparticle targeting and cytocompatibility assays. BV2 cells were stimulated with LPS to induce an activated microglial phenotype before incubation with Cy5-labeled non-targeted or MG1-modified nanoparticles. Cell-associated nanoparticle fluorescence was quantified by flow cytometry and confocal microscopy, and CD86 staining was used to verify activation. For cytocompatibility testing, BV2 and ARPE-19 cells were exposed to free MCC950, MCC950 NPs, or MG1-MCC950 NPs for 24 or 48 h, and viability was measured using CCK-8 assays.

### 5.7 *In vivo* nanoparticle targeting and treatment

Cy5-labeled nanoparticles were administered by intravitreal injection into RVO eyes. Retinas were collected for flow cytometry and confocal imaging, and microglia-associated nanoparticle fluorescence was quantified in CD45^int^CD11b^+^ microglia. For therapy, MCC950 dose screening was performed from 0.01 to 10 mM, and 1 mM was selected for subsequent experiments. RVO mice received PBS, free MCC950, MCC950 nanoparticles, or MG1-MCC950 nanoparticles at equivalent MCC950 doses.

### 5.8 Bulk RNA sequencing and analysis

Whole retinas were collected 48 h after RVO induction. Each experimental condition comprised five animals contributing 10 retinas. The retinas within each condition were pooled before RNA extraction and library preparation, yielding one pooled bulk RNA-seq library for Control and one for RVO. Libraries were prepared from high-quality RNA and sequenced as 150-bp paired-end reads.

Raw reads were trimmed and quality-filtered using fastp, and rRNA-derived sequences were removed using SortMeRNA. Reads were aligned to the GRCm39 mouse reference genome using STAR. For the gene-expression analyses reported here, transcript-level estimated counts and transcripts per million (TPM) were obtained using Kallisto and aggregated to the gene level.

Genes with a combined estimated count of at least 10 across the two libraries were retained for comparison. Gene-wise comparisons were performed using two-sided Fisher's exact tests based on 2 × 2 contingency tables. Each table contained the rounded estimated count for the target gene and the rounded sum of estimated counts for the remaining retained genes in each library. The resulting *P* values were adjusted across all tested genes using the Benjamini–Hochberg procedure. Genes meeting FDR < 0.05 and |log_2_ fold change| ≥ 1 were selected as exploratory candidates.

Gene-level TPM values were transformed as log_2_(TPM + 1) for expression visualization. Functional enrichment analyses and GSEA were performed as described in the pathway-analysis section. Because each condition was represented by one pooled library, between-animal variation in gene expression could not be estimated. Accordingly, the bulk RNA-seq dataset was used for exploratory candidate identification and hypothesis generation.

### 5.9 Single-cell RNA sequencing and analysis

Single-cell RNA sequencing was performed in a separate experimental cohort using retinas collected 48 h after RVO induction. Each experimental condition comprised six animals contributing 12 retinas. The retinas within each condition were pooled to generate one cell suspension and one single-cell RNA-seq library.

Data were processed using Seurat v5.5.0. The input expression matrices contained 6980 Control and 9540 RVO barcodes. Seurat objects were initially constructed with min.cells = 3 and min.features = 200. Subsequent quality control retained cells with more than 200 and fewer than 5000 detected genes and a mitochondrial-transcript proportion below 10%. No additional upper UMI-count threshold was applied. After filtering, 6616 Control cells and 9134 RVO cells were retained.

Expression data were normalized using LogNormalize with a scale factor of 10,000, and 2000 highly variable genes were selected using the “vst” method. The two libraries were integrated using Seurat's anchor-based workflow. Mitochondrial-transcript percentage was regressed out during scaling of the integrated data, and 30 principal components were calculated. The first 15 principal components were used for neighbor identification, clustering at a resolution of 0.05, and uniform manifold approximation and projection (UMAP) visualization. Retinal cell types were annotated using canonical marker genes.

Microglial expression differences between RVO and Control were assessed using log-normalized RNA-assay data with Seurat's FindMarkers function and the Wilcoxon rank-sum test (min.pct = 0.1, logfc.threshold = 0). Exploratory candidate genes were selected using nominal *P* < 0.05 and |log_2_ fold change| ≥ 0.25. Cell composition, functional-state distributions, and pathway-associated scores were summarized descriptively for the two libraries.

### 5.10 Infrared reflectance (IR) fundus and fundus fluorescein angiography (FFA)

IR fundus imaging and FFA were performed using a Heidelberg Spectralis HRA + OCT system after anesthesia and pupillary dilation. IR fundus imaging was used to document retinal vascular morphology, laser-treated sites, and associated retinal changes. For FFA, mice received an intraperitoneal injection of 0.2 mL of 2% sodium fluorescein. Serial angiograms were acquired beginning approximately 2 min after injection.

### 5.11 Optical coherence tomography (OCT)

Spectral-domain OCT was performed using the Heidelberg Spectralis HRA + OCT system in combined IR + OCT mode. Cross-sectional B-scans were acquired at baseline and on days 1–3 after RVO induction in RVO and sham-treated eyes.

To standardize scan location, OCT B-scans in RVO/sham eyes were acquired at the lateral tangent of each laser spot (immediately adjacent to the outer margin of the photocoagulation lesion). For each eye, the mean of three measurements was used as the representative value at each time point. Images with media opacity, severe retinal detachment, or insufficient quality for reliable quantification were excluded.

### 5.12 Electroretinography (ERG)

Full-field electroretinography (ERG) was performed using an Espion electrophysiology system equipped with a Ganzfeld stimulator (Diagnosys LLC, USA). Recordings were obtained on post-induction days 1–3 in the time-course cohort and on post-induction day 2 in the genetic and therapeutic cohorts. Day 2 was selected for the latter cohorts because the time-course experiment showed that retinal electrophysiological impairment was most pronounced at this time point. Before recording, mice were dark-adapted for at least 12 h, anesthetized, and treated with tropicamide eye drops for pupillary dilation. A corneal ring electrode was placed on the corneal surface, a reference electrode was inserted subcutaneously into the cheek, and a ground electrode was positioned at the base of the tail.

Scotopic responses were recorded at flash intensities of 0.001, 0.01, 0.1, 1.0, and 3.0 cd s/m^2^. The a-wave amplitude, b-wave amplitude, and b/a-wave ratio were quantified from responses elicited at 3.0 cd s/m^2^. The a-wave amplitude was measured from the pre-stimulus baseline to the a-wave trough, whereas the b-wave amplitude was measured from the a-wave trough to the b-wave peak. Oscillatory potentials (OPs) were also recorded at 3.0 cd s/m^2^, and total OP amplitude was calculated as the sum of OP1–OP3 amplitudes.

### 5.13 Immunofluorescence

Eyes designated for immunofluorescence were collected on day 2 after RVO induction. Retinal flat mounts were fixed, permeabilized, blocked, and then incubated with primary antibodies against IBA1, GFAP, or RBPMS for 3 days at 4 °C, followed by appropriate Alexa Fluor-conjugated secondary antibodies for 1 h at room temperature. Isolectin GS-IB4 and DAPI were included where indicated. Images were acquired using a Zeiss LSM 880 laser-scanning confocal microscope (Carl Zeiss, Germany).

For retinal section staining, paraffin sections were deparaffinized, rehydrated, and subjected to citrate-buffer antigen retrieval. Sections were blocked and incubated overnight at 4 °C with antibodies against IBA1 together with either NLRP3 or caspase-1, followed by Alexa Fluor-conjugated secondary antibodies for 1 h at room temperature. Nuclei were counterstained with DAPI, and images were acquired using the same confocal microscope. Details of primary antibody are provided in Table S3.

### 5.14 Hematoxylin and eosin (H&E) staining

At 7 days after a single intravitreal injection, the heart, liver, spleen, lungs, and kidneys were collected, fixed, paraffin-embedded, sectioned, and stained with H&E. Sections were examined by light microscopy for evidence of inflammatory cell infiltration, tissue necrosis, hemorrhage, edema, or other overt histopathological abnormalities.

### 5.15 Flow cytometry

Retinas were collected on day 2 after RVO induction and enzymatically dissociated with collagenase II to obtain single-cell suspensions. The suspensions were filtered through a 70-μm cell strainer and washed with ice-cold staining buffer. Cells were incubated with fluorophore-conjugated antibodies against mouse CD45 and CD11b for 30 min at 4 °C in the dark, and a viability dye was used to exclude dead cells. Samples were analyzed using a BD FACSAria system. Debris, doublets, and dead cells were excluded before CD45/CD11b gating. Retinal microglia were defined as CD45^int^ CD11b^+^ cells, and infiltrating myeloid cells as CD45^high^ CD11b^+^ cells.

### 5.16 Western blotting

Retinas were collected on day 2 after RVO induction, and proteins were extracted using RIPA lysis buffer. Equal amounts of protein were separated by sodium dodecyl sulfate–polyacrylamide gel electrophoresis and transferred to membranes. Membranes were incubated with primary antibodies against NLRP3, pro- and cleaved caspase-1, pro- and cleaved IL-1β, pro- and cleaved IL-18, and GAPDH or β-tubulin, followed by the corresponding secondary antibodies. Protein bands were visualized by chemiluminescence and quantified by densitometry using ImageJ.

### 5.17 Quantitative reverse transcription polymerase chain reaction (qRT-PCR)

Retinas were collected on day 2 after RVO induction. Total RNA was reverse-transcribed, and qRT-PCR was performed using SYBR Green. Relative mRNA expression was normalized to *Gapdh* and calculated using the 2^−ΔΔCt^ method.

### 5.18 Statistical analysis

Data are presented as the mean ± SD unless otherwise indicated. For animal experiments, *n* denotes the number of animals per group. For physicochemical characterization, *n* denotes the number of independently prepared formulation batches; technical triplicates were averaged within each batch before statistical analysis. Each sequencing condition was represented by one pooled library. Animals contributing to a pool and individual cells recovered from a library were not counted as independent sequencing replicates.

Statistical analyses were performed using GraphPad Prism, with transcriptomic analyses conducted in R. Depending on the experimental design, comparisons were performed using Welch's unpaired *t*-test, a paired *t*-test, one-way ANOVA followed by Tukey's multiple-comparisons test, two-way ANOVA or two-way repeated-measures ANOVA followed by Šídák's multiple-comparisons test, or Brown–Forsythe and Welch ANOVA followed by Dunnett's T3 multiple-comparisons test, as specified in the corresponding figure legends. Pairwise comparisons were two-sided, and multiplicity-adjusted *P* values were reported where applicable. For independently replicated experiments, *P* < 0.05 was considered statistically significant.

## CRediT authorship contribution statement

**Xiao Xue:** Writing – review & editing, Writing – original draft, Visualization, Validation, Methodology, Investigation, Data curation. **Huimin Jiang:** Writing – review & editing, Visualization, Validation, Investigation, Data curation. **Weiyue Zhang:** Formal analysis, Data curation. **Yifan Zhou:** Investigation, Data curation. **Hui Li:** Validation, Data curation. **Chengbin Zhou:** Resources, Methodology. **Yuxin Chen:** Validation, Investigation. **Wei Ma:** Validation, Investigation. **Yuan Gao:** Visualization, Formal analysis. **Xunming Ji:** Supervision, Resources. **Chen Zhou:** Writing – review & editing, Supervision, Project administration, Conceptualization. **Xuxiang Zhang:** Writing – review & editing, Supervision, Project administration, Funding acquisition, Conceptualization.

## Ethics approval and consent to participate

All animal procedures complied with the ARVO Statement and were approved by the Institutional Animal Care and Use Committee of Capital Medical University (AEEI-2026-139).

## Declaration of competing interest

The authors declare no competing financial interests or personal relationships that could have appeared to influence the work reported in this paper.

## References

[1] Nicholson L., Talks S.J., Amoaku W., Talks K., Sivaprasad S. (2022). Retinal vein occlusion (RVO) guideline: executive summary. Eye.

[2] Scott I.U., Campochiaro P.A., Newman N.J., Biousse V. (2020). Retinal vascular occlusions. Lancet.

[3] Romano F., Lamanna F., Gabrielle P.H., Teo K.Y.C., Battaglia Parodi M., Iacono P. (2023). Update on retinal vein occlusion. Asia Pac J Ophthalmol (Phila).

[4] Chen K.Y., Chan H.C., Chan C.M. (2025). Effectiveness and safety of anti-vascular endothelial growth factor therapies for macular edema in retinal vein occlusion: a systematic review and network meta-analysis of randomized controlled trials. Surv. Ophthalmol..

[5] Cornish E.E., Zagora S.L., Spooner K., Fraser-Bell S. (2023). Management of macular oedema due to retinal vein occlusion: an evidence-based systematic review and meta-analysis. Clin. Exp. Ophthalmol..

[6] Giocanti-Aurégan A., Donati S., Hoerauf H., Allmeier H., Rittenhouse K.D., Machewitz T. (2024). Real-world management of macular edema secondary to retinal vein occlusion with intravitreal aflibercept: 24-month results from the AURIGA observational Study. Ophthalmol Ther.

[7] Dinah C., Dodds M., Lotery A., Salvatore S., Fletcher E., Lake A.V.R. (2026). Treatment patterns and long-term outcomes in anti-VEGF-treated macular oedema secondary to retinal vein occlusion: a retrospective observational study. Eye (Lond).

[8] Munk M.R., Ceklic L., Stillenmunkes R., Chaudhary V., Waheed N., Chhablani J. (2024). Integrated assessment of OCT, multimodal imaging, and cytokine markers for predicting treatment responses in retinal vein occlusion associated macular edema: a comparative review of Anti-VEGF and steroid therapies. Diagnostics.

[9] An Y., Park S.P., Kim Y.K. (2021). Aqueous humor inflammatory cytokine levels and choroidal thickness in patients with macular edema associated with branch retinal vein occlusion. Int. Ophthalmol..

[10] Gong Y., Yang F., Pan X., Zhao J., Yu R., Zhang X. (2025). Aqueous humor cytokine levels in retinal vein occlusion patients with suboptimal response to anti-VEGF therapy and the correlation with OCT imaging biomarkers. Eur. J. Med. Res..

[11] Li X., Cao X., Zhao M., Bao Y. (2022). The changes of irisin and inflammatory cytokines in the age-related macular degeneration and retinal vein occlusion. Front. Endocrinol..

[12] Fan W., Huang W., Chen J., Li N., Mao L., Hou S. (2022). Retinal microglia: functions and diseases. Immunology.

[13] Guo L., Choi S., Bikkannavar P., Cordeiro M.F. (2022). Microglia: key players in retinal ageing and neurodegeneration. Front. Cell. Neurosci..

[14] Zhou L., Xu Z., Lu H., Cho H., Xie Y., Lee G. (2024). Suppression of inner blood-retinal barrier breakdown and pathogenic Müller glia activation in ischemia retinopathy by myeloid cell depletion. J. Neuroinflammation.

[15] Huang Y., Lu J., Zhao L., Fu X., Peng S., Zhang W. (2023). Retinal cell-targeted liposomal ginsenoside Rg3 attenuates retinal ischemia-reperfusion injury via alleviating oxidative stress and promoting microglia/macrophage M2 polarization. Free Radic. Biol. Med..

[16] Tao S., Hu W., Hu Y., Cheng Q., Lin Z., Xu H. (2026). Sfrp2 in Microglia inhibited S100a8-Mediated neuroinflammation and protected neural damage following retinal ischemia-reperfusion. Investig. Ophthalmol. Vis. Sci..

[17] Ebneter A., Kokona D., Schneider N., Zinkernagel M.S. (2017). Microglia activation and recruitment of circulating macrophages during ischemic experimental branch retinal vein occlusion. Investig. Ophthalmol. Vis. Sci..

[18] Jovanovic J., Liu X., Kokona D., Zinkernagel M.S., Ebneter A. (2020). Inhibition of inflammatory cells delays retinal degeneration in experimental retinal vein occlusion in mice. Glia.

[19] Sun C., Li X.X., He X.J., Zhang Q., Tao Y. (2013). Neuroprotective effect of minocycline in a rat model of branch retinal vein occlusion. Exp. Eye Res..

[20] Broz P., Dixit V.M. (2016). Inflammasomes: mechanism of assembly, regulation and signalling. Nat. Rev. Immunol..

[21] Paik S., Kim J.K., Shin H.J., Park E.J., Kim I.S., Jo E.K. (2025). Updated insights into the molecular networks for NLRP3 inflammasome activation. Cell. Mol. Immunol..

[22] Kim J.S., Jun J.H., Lee J., Park S., Kim E., Hwang S.J. (2025). HDAC6 mediates NLRP3 inflammasome activation in the pathogenesis of diabetic retinopathy. Metabolism.

[23] Kong H., Zhao H., Chen T., Song Y., Cui Y. (2022). Targeted P2X7/NLRP3 signaling pathway against inflammation, apoptosis, and pyroptosis of retinal endothelial cells in diabetic retinopathy. Cell Death Dis..

[24] Ortega A.J., Daniel S., Renwick M., Kambhampati P., Thompson K.N., Collier G.E. (2025). Genetic removal of Nlrp3 protects against age-related and R345W Efemp1-induced basal laminar deposit formation. Cell Death Dis..

[25] Ou C., Lin Y., Wen J., Zhang H., Xu Y., Zhang N. (2024). Roflumilast Attenuates microglial senescence and retinal inflammatory neurodegeneration post retinal ischemia reperfusion injury through inhibiting NLRP3 inflammasome. Investig. Ophthalmol. Vis. Sci..

[26] Coll R.C., Hill J.R., Day C.J., Zamoshnikova A., Boucher D., Massey N.L. (2019). MCC950 directly targets the NLRP3 ATP-hydrolysis motif for inflammasome inhibition. Nat. Chem. Biol..

[27] Terashima T., Ogawa N., Nakae Y., Sato T., Katagi M., Okano J. (2018). Gene therapy for neuropathic pain through siRNA-IRF5 gene delivery with homing peptides to microglia. Mol. Ther. Nucleic Acids.

[28] Li H., Guan X., Liu L., Lu C., Zhang W., Zhou Y. (2026). Cerebral venous outflow insufficiency: a Study on symptoms and venous stenosis classification. MedComm.

[29] Wei S., Li Y., Yu X., Ding R., Meng G. (2026). Progress on the mechanism of pathological injury secondary to venous stroke and their therapeutic strategies: a narrative review. Brain Circ.

[30] Zhou Y., Qi J., Liu H., Liang S., Guo T., Chen J. (2023). Increased intraocular inflammation in retinal vein occlusion is independent of circulating immune mediators and is involved in retinal oedema. Front. Neurosci..

[31] Zhang N., Tian T., Wang Z., He X., Cao W., Zhang W. (2025). ATF3 prevents retinal ganglion cell apoptosis and mitigates microglia-mediated neuroinflammation in retinal ischemia-reperfusion injury. Front. Immunol..

[32] Mi R., Cheng H., Chen R., Bai B., Li A., Gao F. (2024). Effects and mechanisms of long-acting glucagon-like peptide-1 receptor agonist semaglutide on microglia phenotypic transformation and neuroinflammation after cerebral ischemia/reperfusion in rats. Brain Circ.

[33] Gao S., Li N., Lin Z., Zhong Y., Wang Y., Shen X. (2024). Inhibition of NLRP3 inflammasome by MCC950 under hypoxia alleviates photoreceptor apoptosis via inducing autophagy in Müller glia. FASEB J..

[34] Ren K., Li X. (2025). MCC950 targets the ROS-NEK7-NLRP3 axis to improve type 2 diabetic retinopathy. Sci. Rep..

[35] Baig M.S., Karade S.K., Ahmad A., Khan M.A., Haque A., Webster T.J. (2024). Lipid-based nanoparticles: innovations in ocular drug delivery. Front. Mol. Biosci..

