# Supplementary material for: Microglia-targeted delivery of MCC950 suppresses NLRP3–IL-18-mediated neuroinflammation and retinal injury in retinal vein occlusion
Source: Bioact Mater. 2026 Oct 22;69:850–67. doi: 10.1016/j.bioactmat.2026.10.022 (PMC13715420; doi:10.1016/j.bioactmat.2026.10.022)
Supplement: Supplementary file 1 [file mmc3.docx]

**Supplementary information**

**Microglia-Targeted Delivery of MCC950 Suppresses NLRP3-IL-18-Mediated**

**Neuroinflammation and Retinal Injury in Retinal Vein Occlusion**

Xue et al.

Figure S1-S11

Table S1-S5

**Figure S1. Single-cell RNA-sequencing quality-control metrics, cell-type overlap, and microglial-state composition.** (A–C) Violin plots showing the number of detected genes per cell, unique molecular identifier counts per cell, and the percentage of mitochondrial reads in control and RVO retinal single-cell datasets, respectively. (D) Separate and overlaid UMAP visualizations showing the distribution and overlap of control- and RVO-derived cells across the annotated retinal cell types. (E) Stacked bar plot showing the relative proportions of annotated retinal cell types in the control and RVO groups. (F) Stacked bar plot showing the relative composition of homeostatic, inflammatory-response, interferon-response, phagocytic/complement, stress/metabolic, and other microglial states in control and RVO retinas. RVO, retinal vein occlusion; UMAP, uniform manifold approximation and projection; UMI, unique molecular identifier.

**Figure S2. Flow-cytometry gating strategy for retinal microglia.** (A) Representative sequential gating workflow showing exclusion of debris by FSC/SSC, singlet selection, DAPI-based exclusion of dead cells, and identification of retinal microglia within the CD45^int^CD11b^+^ population.

**Figure S3. Constitutive global deletion of *Nlrp3* suppresses inflammasome signaling and attenuates microglial remodeling after RVO.** (A) Genotyping PCR showing the wild-type (331 bp) and *Nlrp3*-knockout (628 bp) alleles. (B–D) Reverse transcription–quantitative PCR analysis of *Nlrp3*, *Il1b*, and *Il18* mRNA expression, respectively, in WT-sham, WT-RVO, *Nlrp3*^-/-^-sham, and *Nlrp3*^-/-^-RVO retinas. (E) Retinal flat-mount immunofluorescence of isolectin GS-IB4-labeled vasculature and IBA1^+^ microglia in the deep capillary plexus. (F, G) Retinal-section immunofluorescence showing NLRP3–IBA1 and caspase-1–IBA1 colocalization, respectively, in *Nlrp3*^-/-^ retinas. Insets show representative regions at higher magnification. (H) Western blot analysis of NLRP3 and the pro- and cleaved forms of caspase-1, IL-1β, and IL-18. (I–L) Densitometric quantification of NLRP3, cleaved caspase-1, cleaved IL-1β, and cleaved IL-18, respectively. Scale bars in E–G, 50 μm. Data in B–D and I–L are presented as the mean ± SD; *n* = 6 animals per group. Statistical comparisons were performed using two-way ANOVA followed by Šídák’s multiple-comparisons test. ns, not significant; ****P* < 0.001. RVO, retinal vein occlusion; WT, wild type.

**Figure S4. Constitutive global deletion of *Il18* attenuates retinal thickening, electrophysiological impairment, and microglial activation after RVO.** (A) Representative fundus fluorescein angiography (FFA), infrared reflectance (IR) fundus, and optical coherence tomography (OCT) images from WT-sham, WT-RVO, *Il18*^-/-^-sham, and *Il18*^-/-^-RVO eyes. (B–E) Quantification of whole retinal, inner retinal, nerve fiber layer–ganglion cell layer (NFL–GCL), and inner nuclear layer (INL) thickness, respectively. (F) Representative scotopic electroretinography (ERG) and oscillatory potential (OP) waveforms. (G–I) Quantification of b wave amplitude, b/a wave ratio, and total OP amplitude, respectively, from responses elicited at 3.0 cd·s/m². (J, K) Representative flow-cytometry plots and quantification of CD45^int^CD11b^+^ retinal microglia. (L) Retinal flat-mount immunofluorescence of isolectin GS-IB4-labeled vasculature and IBA1^+^ microglia. (M–O) Quantification of IBA1^+^ area and fold changes in microglial branch length and endpoint number, respectively. (P) Genotyping PCR showing the WT (332 bp) and *Il18*-knockout (415 bp) alleles. Scale bars in L, 50 μm. Data in B–E, G–I, K, and M–O are presented as the mean ± SD; *n* = 6 animals per group. Statistical comparisons were performed using two-way ANOVA followed by Šídák’s multiple-comparisons test. **P* < 0.05, ***P* < 0.01, and ****P* < 0.001. RVO, retinal vein occlusion; WT, wild type.

**Figure S5.** **Effect of *Il18* deficiency on microglial morphology in the deep retinal capillary plexus after RVO.** (A) Representative retinal flat-mount immunofluorescence showing isolectin GS-IB4-labeled vasculature and IBA1-positive microglia in the deep capillary plexus of WT-sham, WT-RVO, *Il18*^-/-^-sham, and *Il18*^-/-^-RVO retinas. Scale bars, 50 μm. RVO, retinal vein occlusion; WT, wild type.

**Figure S6. Synthesis and characterization of DSPE–PEG–MG1.** (A) Synthetic scheme for the conjugation of MG1 (CHHSSSARC) with DSPE–PEG–NHS. (B) ^1^H NMR spectra of MG1 and DSPE–PEG–MG1 recorded at 400 MHz in DMSO-*d₆*. The boxed regions at approximately δ 7.0–8.2 ppm indicate peptide-associated signals.

**Figure S7. MG1 functionalization enhances nanoliposome association with activated microglia *in vitro* and promotes preferential microglial localization *in vivo*.** (A) Western blot showing increased NLRP3 expression in BV2 cells following lipopolysaccharide (LPS) stimulation. (B, C) Representative flow-cytometry histograms and quantification of nanoparticle-associated Cy5 fluorescence in unstimulated BV2 cells and LPS-stimulated BV2 cells exposed to non-targeted NPs or MG1-NPs. (D) Representative confocal images showing CD86 immunoreactivity and MG1-NP-associated fluorescence in unstimulated and LPS-stimulated BV2 cells. (E, F) Quantification of MG1-NP-associated fluorescence and CD86 fluorescence, respectively. (G, H) Representative flow-cytometry histograms and quantification of nanoparticle-associated fluorescence in retinal microglia isolated from RVO eyes following intravitreal administration of non-targeted NPs or MG1-NPs. (I) Representative retinal-section images showing nanoparticle-associated fluorescence, IBA1^+^ microglia, and DAPI-stained nuclei after intravitreal administration of non-targeted NPs or MG1-NPs into RVO eyes. (J) Quantification of the proportion of IBA1^+^ microglia associated with detectable nanoparticle fluorescence. Scale bars, 10 μm in D and 50 μm in I. Data are presented as the mean ± SD; *n* = 3 per group in C and *n* = 6 per group in E, F, H, and J. Statistical comparisons were performed using one-way ANOVA followed by Tukey’s multiple-comparisons test in C and Welch’s unpaired *t*-test in E, F, H, and J. ****P* < 0.001 and *****P* < 0.0001. LPS, lipopolysaccharide; NPs, nanoparticles; RVO, retinal vein occlusion.

**Figure S8. Preferential localization of MG1-NPs to retinal microglia *in vivo*.** (A) Representative flow-cytometric gating strategy for viable singlets, retinal microglia (CD45^int^CD11b^+^), and infiltrating myeloid cells (CD45^high^CD11b^+^), followed by measurement of MG1-NPs-associated Cy5 fluorescence. (B) Representative mode-normalized histogram overlay showing the distribution of MG1-NP-associated fluorescence in retinal microglia and infiltrating myeloid cells. (C) Paired quantification of mean Cy5 fluorescence intensity in the two cell populations. (D) Representative orthogonal projections of confocal z-stacks showing the spatial association of MG1-NP fluorescence with IBA1^+^ microglia, GFAP^+^ glial cells, and RBPMS^+^ retinal ganglion cells. (E) Representative retinal flat-mount images showing MG1-NPs fluorescence and the indicated cell-type markers. MG1-NPs are shown in green, cellular markers in red, and DAPI-stained nuclei in blue. Scale bars, 50 μm. (F) Quantification of the proportion of cells associated with detectable MG1-NPs fluorescence within each marker-defined retinal cell population. Data in C and F are presented as the mean ± SD; *n* = 6 animals per group. The comparison in C was performed using a two-sided paired *t*-test. Comparisons in F were performed using Brown–Forsythe and Welch ANOVA followed by Dunnett’s T3 multiple-comparisons test. ****P* < 0.001. GFAP, glial fibrillary acidic protein; NPs, nanoparticles; RBPMS, RNA-binding protein with multiple splicing; RGCs, retinal ganglion cells.

**Figure S9. Screening of intravitreal MCC950 concentrations for inhibition of retinal inflammasome signaling after RVO.** (A) Representative western blots of NLRP3, cleaved caspase-1, cleaved IL-18, and cleaved IL-1β in non-RVO retinas, vehicle-treated RVO retinas, and RVO retinas treated with increasing concentrations of MCC950 (0.01, 0.1, 1, and 10 mM). A concentration of 1 mM was selected for subsequent therapeutic experiments because it produced marked inflammasome inhibition without requiring the highest concentration tested. RVO, retinal vein occlusion.

**Figure S10. MG1-MCC950 nanoliposomes suppress microglial activation and retinal inflammasome signaling after RVO.** (A) Representative flow-cytometry plots showing CD45^int^CD11b^+^ retinal microglia in RVO eyes treated with PBS, free MCC950, MCC950 NPs, or MG1-MCC950 NPs. (B) Representative retinal flat-mount immunofluorescence showing isolectin GS-IB4-labeled vasculature and IBA1-positive microglia in the deep capillary plexus after each treatment. Scale bars, 50 μm. (C) Western blot analysis of NLRP3 and the pro- and cleaved forms of caspase-1, IL-1β, and IL-18. (D–G) Densitometric quantification of NLRP3, cleaved caspase-1, cleaved IL-1β, and cleaved IL-18, respectively. Data in D–G are presented as the mean ± SD; *n* = 6 animals per group. Statistical comparisons were performed using one-way ANOVA followed by Tukey’s multiple-comparisons test. ****P* < 0.001. NPs, nanoparticles; PBS, phosphate-buffered saline; RVO, retinal vein occlusion.

**Figure S11. Preliminary histological biosafety and cytocompatibility assessment of MG1-MCC950 nanoliposomes.** (A) Representative hematoxylin and eosin (H&E)-stained sections of the heart, liver, spleen, lung, and kidney collected 7 days after a single intravitreal administration of PBS, free MCC950, MCC950 NPs, or MG1-MCC950 NPs. No overt treatment-associated histopathological abnormalities were observed under the conditions examined. Scale bars, 50 μm for heart, 200 μm for liver, and 100 μm for spleen, lung, and kidney. *n* = 3 animals per group. (B) Cell Counting Kit-8 analysis of BV2 and ARPE-19 cell viability following exposure to free MCC950, MCC950 NPs, or MG1-MCC950 NPs at 1, 10, or 100 μM for 24 or 48 h. Data are presented as the mean ± SD; *n* = 6 per condition. NPs, nanoparticles; PBS, phosphate-buffered saline.

**Table S1. Physicochemical characterization of nanoparticle formulations**

| **Formulation** | **Particle size**  **(nm)** | **PDI** | | **Zeta potential (mV)** | **MCC950 loading (w/w, %)** | **EE (%)** |
| --- | --- | --- | --- | --- | --- | --- |
| NPs | 136.25 ± 3.64 | 0.236 ± 0.017 | −21.03 ± 1.11 | | — | — |
| MG1-NPs | 136.74 ± 7.13 | 0.251 ± 0.017 | −21.68 ± 0.44 | | — | — |
| MCC950 NPs | 138.23 ± 1.70 | 0.217 ± 0.020 | −19.27 ± 1.72 | | 7.16 ± 0.65 | 89.44± 0.83 |
| MG1-MCC950 NPs | 138.50 ± 2.05 | 0.263 ± 0.020 | −24.19 ± 2.08 | | 6.23± 0.59 | 82.86± 0.62 |

Data are presented as mean ± SD from three independently prepared batches (*n* = 3). —, not applicable; PDI, polydispersity index; EE, encapsulation efficiency.

**Table S2. Primer sequences for mouse genotyping and qRT-PCR**

| **Mouse genotyping** | |
| --- | --- |
| *Il18* WT F | 5’-GGCAGCAAGCACTCTTAACC-3’ |
| *Il18* Common R | 5’-ACAAACCCTCCCCACCTAAC-3’ |
| *Il18* Mutant F | 5’-AATTGGCCAATGACAAGACG-3’ |
| *Nlrp3* KO A2 | 5’-TCGCCTTCTTGACGAGTTCT-3’ |
| *Nlrp3* WT B2 | 5’-CTGTGATGGATGCCAGATTG-3’ |
| *Nlrp3* Common C2 | 5’-GCACGTTCACCCTACCCTTA-3’ |
| **qRT-PCR primers** | |
| Mouse-*Nlrp3*-forward | 5’-ATTACCCGCCCGAGAAAGG-3’ |
| Mouse-*Nlrp3*-reverse | 5’-TCGCAGCAAAGATCCACACAG-3’ |
| Mouse-*Il1b*-forward | 5’-GGCTGGACTGTTTCTAATGC-3’ |
| Mouse-*Il1b* -reverse | 5’-ATGGTTTCTTGTGACCCTGA-3’ |
| Mouse-*Il18*-forward | 5’-GTCAGAAGACTCTTGCGTCAACT-3’ |
| Mouse-*Il18*-reverse | 5’-TATTCCGTATTACTGCGGTTGT-3’ |
| Mouse-*Gapdh*-forward | 5’-CATCACTGCCACCCAGAAGACTG-3’ |
| Mouse-*Gapdh*-reverse | 5’-ATGCCAGTGAGCTTCCCGTTCAG-3’ |

**Table S3. Antibodies and experimental conditions used in this study**

| Protein | Species | Application | Manufacturer | Catalog No. | Dilution |
| --- | --- | --- | --- | --- | --- |
| anti-NLRP3 | Mouse | WB | Adipogen | AG-20B-0014 | 1:5000 |
|  |  | IF |  |  | 1:100 |
| anti-caspase-1 | Mouse | WB | Santa Cruz | sc-392736 | 1:1000 |
|  |  | IF |  |  | 1:100 |
| anti-IL-1β | Rabbit | WB | Proteintech | 16806-1-AP | 1:5000 |
| anti-IL-18 | Rabbit | WB | Proteintech | 10663-1-AP | 1:8000 |
| anti-GAPDH | Rabbit | WB | Affinity | AF7021 | 1:20000 |
| Anti-β-Tubulin | Mouse | WB | Abmart | M20005 | 1:2000 |
| anti-IBA-1 | Rabbit | IF  (Flat-mount) | Wako | 019-19741 | 1:500 |
|  |  | IF  (paraffin section) | Abcam | AB178847 | 1:300 |
| anti-RBPMS | Rabbit | IF  (Flat-mount) | Proteintech | 85086-5-RR | 1:300 |
| anti-GFAP | Rabbit | IF  (Flat-mount) | Proteintech | 16825-1-PBS | 1:100 |
| anti-CD86-APC | Mouse | FC | BioLegend | 105011 | 1:100 |
| Anti-CD11b-FITC | Rat | FC | BioLegend | 101206 | 1:100 |
| Anti-CD45-PE/Cy7 | Rat | FC | BioLegend | 103114 | 1:100 |

**Table S4. Bulk RNA-seq gene expression statistics**

The complete gene-level results, including unadjusted *P* values, Benjamini–Hochberg-adjusted FDR values, and log₂ fold changes, are provided in a separate Excel file.

**Table S5. Single-cell RNA-seq differential expression statistics for microglia**

The complete microglial gene-level results, including log₂ fold changes, unadjusted *P* values, and multiple-testing-adjusted *P* values, are provided in a separate Excel file.
